# Identification and evaluation of tumor pyroptosis-associated antigens for design a vaccine candidate against lung cancer

**DOI:** 10.1038/s41598-024-84792-4

**Published:** 2026-03-19

**Authors:** Truc Ly Nguyen, Heebal Kim

**Affiliations:** 1 Department of Agricultural Biotechnology and Research Institute of Agriculture and Life Sciences, Seoul National University, Seoul, 08826 Republic of Korea; 2 Interdisciplinary Program in Bioinformatics, Seoul National University, Seoul, 08826 Republic of Korea; 3 eGnome, Inc., Seoul, 05836 Republic of Korea

**Keywords:** Lung cancer, Multi-epitope vaccine, Pyroptosis-associated antigens, Immunoinformatics, Computational vaccine design, Molecular dynamics simulation, Non-small-cell lung cancer, Protein structure predictions, Peptide vaccines

## Abstract

Lung cancer is the leading cause of cancer-related mortality worldwide, emphasizing the need for innovative therapeutic strategies. This study utilized immunoinformatics and structural bioinformatics approaches to design and evaluate a multi-epitope vaccine targeting pyroptosis-associated antigens (CARD8, NAIP, NLRP1, and NLRP3), which are implicated in lung cancer immunology. Fifteen T-cell and B-cell epitopes were identified, analyzed for their antigenicity, non-toxicity, non-allergenicity, and immune-stimulatory potential, and optimized to construct a vaccine with suitable adjuvants and linkers. The vaccine demonstrated high antigenicity, solubility, and stability, as validated through physicochemical analyses. Its three-dimensional structure was modeled, refined, and validated using molecular modeling approaches. Molecular docking studies revealed stable and strong interactions between the vaccine and key immune receptors (TLR2, TLR4, TLR5, TLR3, TLR7, and TLR8), indicating its potential to activate both innate and adaptive immunity. Molecular dynamics simulations confirmed the vaccine’s structural stability and solvent accessibility over 100 ns across ten replicas. Immune simulations demonstrated strong immunogenic responses, including elevated antibody titers, memory cell populations, and cytokine production. Codon optimization and in silico cloning further ensured efficient expression in *Escherichia coli*, facilitating experimental application. These findings underscore the vaccine’s promise as a therapeutic candidate against lung cancer, warranting further in vitro and in vivo investigations.

## Introduction

Lung cancer is one of the most prevalent and lethal cancers worldwide, originating in the tissues of the lungs, typically within the cells lining the air passages. It is broadly classified into two main types: non-small cell lung cancer (NSCLC), which accounts for about 85% of all cases, and small cell lung cancer (SCLC)^1^. According to the World Health Organization (WHO), lung cancer is the leading cause of cancer-related deaths globally, responsible for approximately 1.8 million deaths annually. In 2022, GLOBOCAN estimated that lung cancer was the most commonly diagnosed cancer, with nearly 2.5 million new cases, representing one in every eight cancer diagnoses globally^2^. In the United States alone, the American Cancer Society estimates that in 2024, there will be about 234,580 new cases of lung cancer and around 125,070 deaths^3^. These alarming statistics underscore the urgent need for effective prevention, early detection, and treatment strategies. The treatment of lung cancer depends on the type, stage, and overall health of the patient, with common treatments including surgery, targeted therapy, radiation therapy, chemotherapy, and immunotherapy^4^.

Vaccines play a critical role in preventing various diseases, including certain cancers. While traditional vaccines prevent infections that can lead to cancer (e.g., HPV vaccine for cervical cancer)^5^, cancer vaccines aim to stimulate the immune system to recognize and destroy cancer cells. Currently, no FDA-approved vaccines specifically target lung cancer, though several are under development and in clinical trials. These vaccines aim to prompt the immune system to recognize and attack lung cancer cells. For example, the Center for Molecular Immunology (CIM) in Havana, Cuba, has developed CIMAvax-EGF, a vaccine that targets the epidermal growth factor (EGF), a protein crucial for cancer cell growth^6^. CIMAvax-EGF works by preventing EGF from binding to its receptor (EGFR) on cancer cells, thus inhibiting their proliferation and leading to their eventual death^7^. Another vaccine candidate, LungVax, aims to prevent lung cancer in high-risk individuals, particularly current or former smokers aged 55–74, by training the immune system to recognize neoantigens (mutant proteins on abnormal lung cells) thereby targeting and eliminating these cancerous cells^8^. OSE2101 is a T-cell-specific immunotherapy designed to induce cytotoxic T lymphocytes (CTLs) against five tumor-associated antigens (TAAs) commonly overexpressed in NSCLC: HER-2/neu, CEA, MAGE 2, MAGE 3, and p53^9^. This therapeutic vaccine contains nine synthetic peptides derived from these TAAs, which are presented by the human leukocyte antigen (HLA)-A2 phenotype, found in up to 45% of the population. Additionally, a tenth pan-DR peptide stimulates the T-helper lymphocyte immune response. In a study comparing OSE2101 to chemotherapy in NSCLC patients who developed resistance to immunotherapy, OSE2101 improved survival and demonstrated better safety^10^. A promising new approach involves mRNA vaccines for NSCLC, such as BI 1361849^11^, which contains six modified mRNAs encoding different NSCLC-associated antigens. These mRNA-derived vaccines have the potential to stimulate both humoral and cellular responses against NSCLC cells, with clinical trials ongoing (NCT03164772). The antigens encoded by BI 1,361,849 are frequently overexpressed in NSCLC and minimally present in healthy cells, suggesting a strong antitumor effect^12^.

Immunoinformatics, the intersection of immunology and bioinformatics, plays a pivotal role in modern vaccine development by enabling the prediction of potential antigens, immune responses, and designing more effective vaccines. In this study, a vaccine candidate was designed using tumor pyroptosis-associated antigens for lung cancer. Previous research identified CARD8, NAIP, NLRP1, and NLRP3, which are associated with tumor pyroptosis, as promising targets for lung adenocarcinoma vaccine development^13^. These proteins were analyzed to identify T-cell and B-cell epitopes. The selected epitopes, which met various immunogenic criteria, were incorporated into a multi-epitope vaccine construct with suitable adjuvants and linkers. The structural modeling of the vaccine was followed by molecular dynamics simulation analysis to validate its stability. Finally, the vaccine’s potential to induce an immune response was evaluated using computational immune simulation.

## Results

### Sequence retrieval

The UniProt IDs of the proteins CARD8, NAIP, NLRP1, and NLRP3 are Q9Y2G2, Q13075, Q9C000, and Q96P20, respectively. The sequences of these proteins are provided in Tables S1–S4.

### Prediction and analysis of the T-cell and B-cell epitopes

A total of 334 strong-binding MHC-II epitopes and 724 strong-binding MHC-I epitopes were predicted from the selected tumor pyroptosis-associated antigens using NetMHCIIpan-4.3 and NetMHCpan-4.1, respectively (Supplementary Data Sheets). Additionally, 37 B-cell epitopes were identified using the BepiPred-3.0 server (Supplementary Data Sheets). After further analysis, 15 epitopes were selected for the lung cancer vaccine design, comprising 3 B-cell, 4 MHC-II, and 8 MHC-I epitopes (Table 1). These selected epitopes were confirmed to be antigenic (with VaxiJen scores above 1.1, except for B-cell epitopes), non-allergenic, non-toxic, and capable of inducing interleukin-4 (IL-4), interleukin-10 (IL-10), and interferon-gamma (IFN-γ). The T-cell epitopes in the final vaccine design are predicted to provide population coverage of 77.21% globally (Table 2).

**Table 1:** Final epitopes chosen for vaccine design, including their binding MHC alleles, binding affinity, and VaxiJen scores.

Type of epitope	Protein	Epitopes	Binding MHC alleles	Binding affinity	VaxiJen score^a^
B-cell	NAIP	ELEEEEQKERAKMQKGYNSQMRS	NA	NA	0.8518
		ESLENISENDDYLKH	NA	NA	0.9536
	NLRP3	QQMESGKSLAQTSKTT	NA	NA	0.9124
MHC-II	NAIP	GKMRYQEEEARLASF	DRB1_0101	18.94	1.1007
		KYDIRVKNLKSRLRG	DRB1_1301	6.31	1.3449
	NLRP1	GLYQALKETHPHLIM	DRB1_0701	7.51	1.1467
	NLRP3	EGLLHPDCKLQVLEL	DRB1_0301	26.37	1.1553
MHC-I	CARD8	MVLEHPARV	HLA-A*02:01	30.75	1.1531
	NAIP	LPRLIRLNM	HLA-B*07:02, HLA-B*08:01	8.71	1.4736
	NLRP1	YLIPSDCSI	HLA-A*02:01	12.62	1.191
		RPRCLLETL	HLA-B*07:02	4.11	1.1913
		HPACKLIRL	HLA-B*07:02, HLA-B*08:01	40.3	1.9683
	NLRP3	EQARWVEVL	HLA-B*39:01	56.18	1.1178
		VKDKKDETL	HLA-B*39:01	2936.06	1.3621
		KAKAKKLQI	HLA-B*08:01	1287.77	1.4412

^a^VaxiJen score above 1.1 for MHC epitopes, excepting B-cell epitopes.

**Table 2:** Population coverage analysis results from IEDB, with global population coverage estimated at 77.21%.

Population/area	Class combined		
	Coverage^a^ (%)	Average_hit^b^	pc90^c^
Central Africa	54.68	1.11	0.22
Central America	19.33	0.20	0.12
East Africa	58.28	1.24	0.24
East Asia	49.94	1.12	0.20
Europe	87.56	2.89	0.80
North Africa	69.65	1.52	0.33
North America	80.88	2.21	0.52
Northeast Asia	34.92	0.63	0.15
Oceania	30.65	0.76	0.14
South Africa	57.34	1.12	0.23
South America	46.63	0.96	0.19
South Asia	63.33	1.05	0.27
Southeast Asia	35.21	0.67	0.15
Southwest Asia	53.71	1.22	0.22
West Africa	52.29	1.17	0.21
West Indies	68.74	1.82	0.32
World	77.21	2.10	0.44
Average	55.31	1.28	0.28
Standard deviation	17.95	0.64	0.17

^a^Projected population coverage.

^b^Average number of epitope hits/HLA combinations recognized by the population.

^c^Minimum number of epitope hits/HLA combinations recognized by 90% of the population.

### Designing the lung cancer vaccine candidate and analysis

**Fig. 1:**
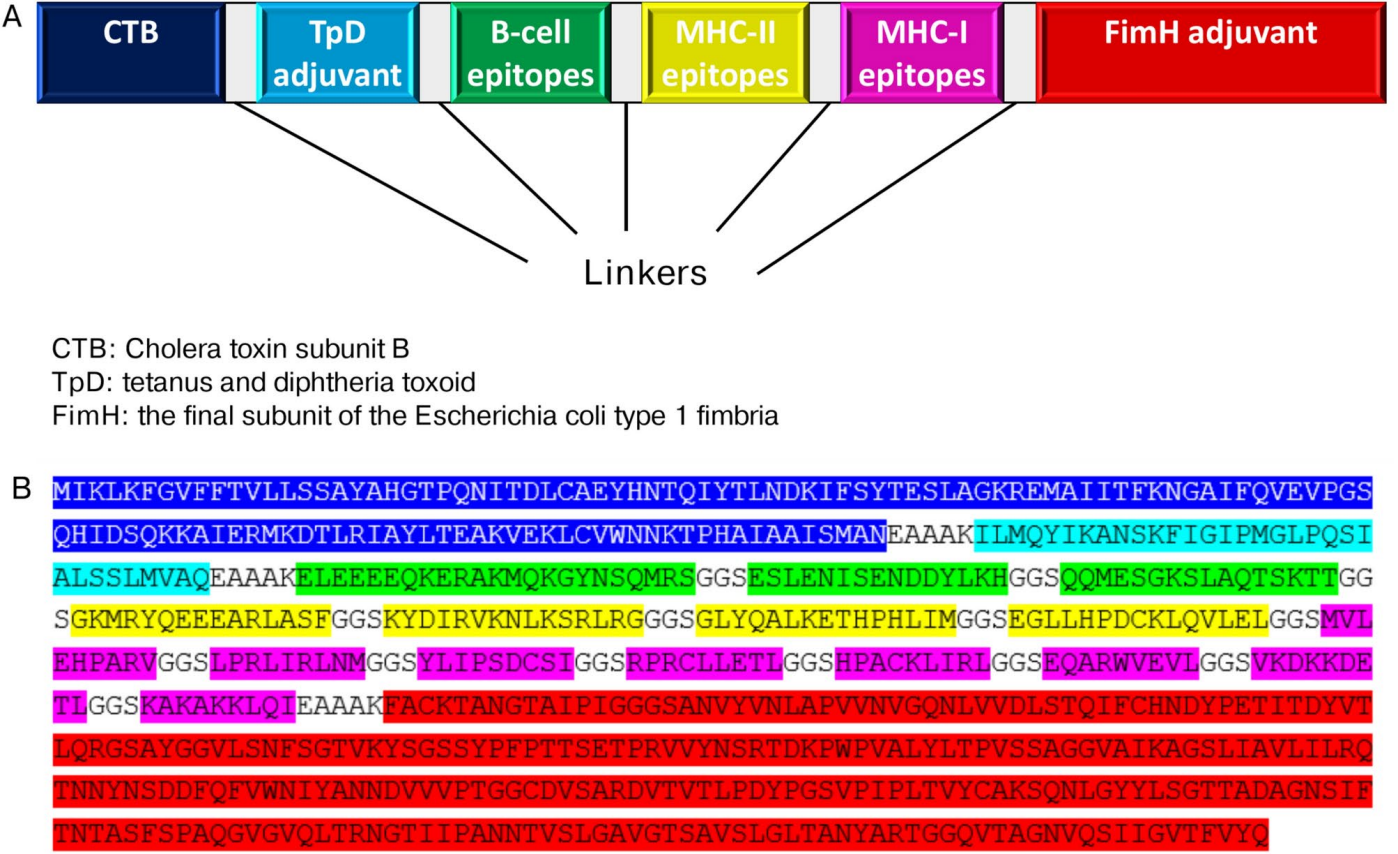
Schematic representation (**A**) and sequence (**B**) of the designed vaccine with adjuvants, linkers, and epitopes sequentially and appropriately.

Based on the results in Table 1, the 15 selected epitopes (3 B-cell, 4 MHC-II, and 8 MHC-I epitopes) were connected using GGS linkers, while adjuvants were linked using EAAAK linkers. The resulting vaccine sequence is depicted in Fig. 1.

The designed vaccine consists of 678 amino acids and exhibits favorable physicochemical properties. The instability index of 35.98 classifies the vaccine as stable, and the antigenicity score (VaxiJen score: 0.6472) confirms its potential to elicit an immune response. Additionally, the vaccine was predicted to be non-allergenic and non-toxic, making it a suitable candidate for therapeutic applications. The solubility of the vaccine formulation was evaluated using the SoluProt v1.0 server, which predicts the likelihood of soluble protein expression in *Escherichia coli*. The solubility score of 0.904, significantly exceeding the threshold of 0.5, indicates that the vaccine candidate is highly soluble and suitable for efficient expression in *E. coli*. This suggests a strong potential for successful production in bacterial expression systems. Further evaluation of the vaccine’s physicochemical properties, including its theoretical isoelectric point (pI: 8.87), aliphatic index (85.60), and hydrophilicity (GRAVY score: − 0.162), reveals its thermodynamic stability and hydrophilic nature. These features collectively support the vaccine’s potential for effective expression, stability, and immunogenicity. Detailed physicochemical properties are summarized in Table 3.

**Table 3:** Physicochemical properties of the vaccine.

Features	Assessment	Remark
Number of amino acids	678	–
Molecular weight	72460.42	Average
Theoretical pI	8.87	Basic nature
Aliphatic index (AI)	85.60	Thermostable
Instability index (II)	35.98	Stable
Grand average of hydropathicity (GRAVY)	− 0.162	Hydrophilic
Solubility	0.904 (SoluProt v1.0)	Soluble
Estimated half-life (mammalian reticulocytes, in vitro)	30 h	Satisfactory
Estimated half-life (yeast cells, in vivo)	> 20 h	Satisfactory
Estimated half-life (*Escherichia coli*, in vivo)	> 10 h	Satisfactory
Antigenicity	0.6472 (VaxiJen v2.0, threshold 0.4)	Antigenic
Allergenicity	Non-allergen (AllerTop v2.0)	Nonallergen
Toxicity	ToxicPred	Nontoxic

### Structure prediction, refinement, and validation of the vaccine candidate

**Fig. 2:**
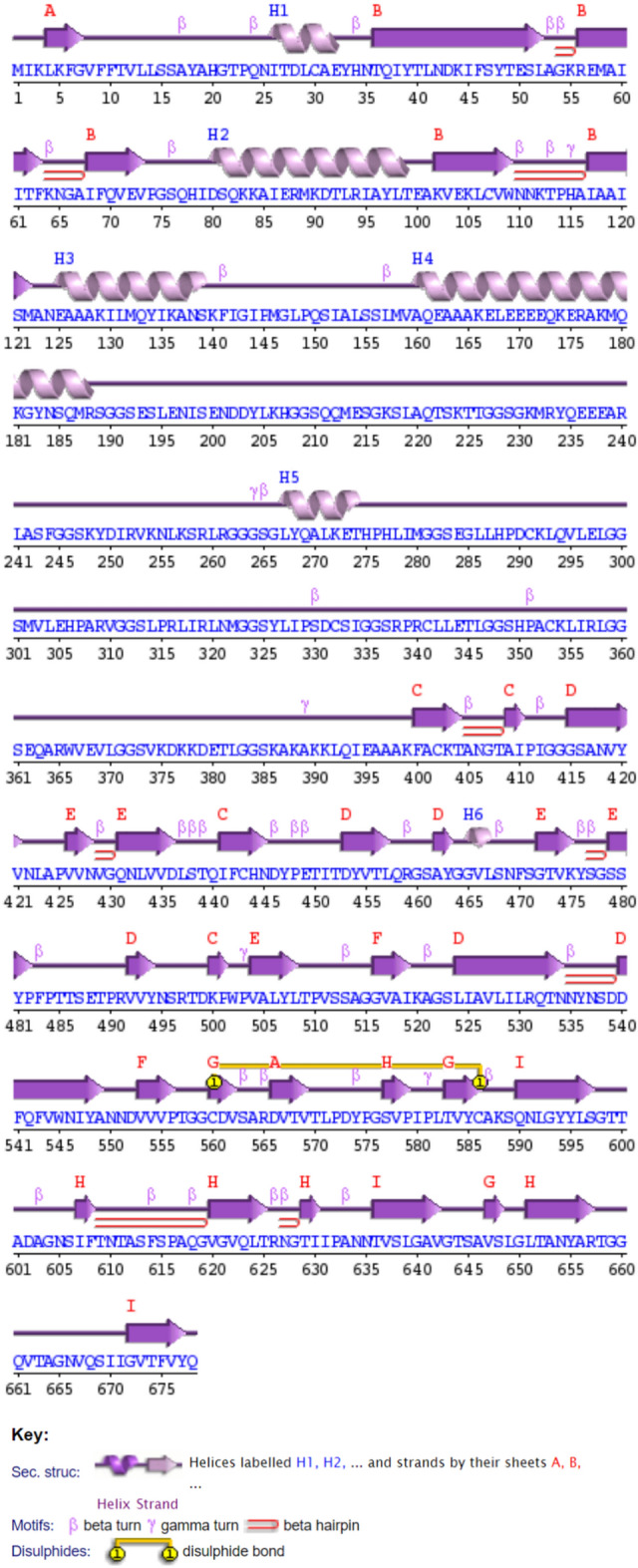
Secondary structure prediction of the designed vaccine obtained from PDBsum server.

The vaccine’s secondary structure was predicted using PDBsum, showing that out of 678 residues, 189 are in beta strands, 79 in alpha helices, and 164 in beta turns (Fig. 2).

For 3D structure prediction, SWISS-MODEL, I-TASSER, and AlphaFold2 were used. Initially, SWISS-MODEL yielded a low GMQE score (0.40) (Fig. S1), and I-TASSER was subsequently used to generate the top five 3D models (Table S5). Among them, model 1 (Fig. S2A) was selected for refinement, but its Ramachandran plot showed only 62.3% of residues in favored regions (Fig. S2B). The GalaxyRefine server was then used to refine the structure, yielding five refined models (Table S6). According to Table S6, model 3 (Fig. S2C) was identified as having the highest GDT-HA score (0.9399) and the lowest RMSD (0.448). Nonetheless, the PROCHECK program confirmed the stereochemical quality of this model structure with only 82.9% of residues in the most favored regions (Fig S2D). A good quality model should include more than 90% of residues in the most favorable regions. Therefore, AlphaFold2 was employed to generate five models (Fig. S3A) and picked the top-scoring prediction as the best model (Fig. S3B). However, the Ramachandran plot analysis revealed only 66.8% of residues in the most favored regions (Fig. S3C), resulting in an additional structural refinement that was performed, which generated five refined models and their features as shown in Table 4.

**Table 4:** Structure Information obtained from GalaxyRefine output page.

Model	GDT-HA	RMSD	MolProbity	Clash score	Poor rotamers	Rama favoured (%)
Initial	1.0000	0.000	3.443	28.7	7.7	71.4
MODEL 1	0.8931	0.660	1.630	7.5	0.2	96.6
MODEL 2	0.8931	0.663	1.667	7.4	0.0	96.2
MODEL 3	0.8857	0.693	1.589	7.0	0.4	96.7
MODEL 4	0.8960	0.656	1.649	7.3	0.4	96.3
MODEL 5	0.8979	0.629	1.679	7.4	0.4	96.0

**Fig. 3:**
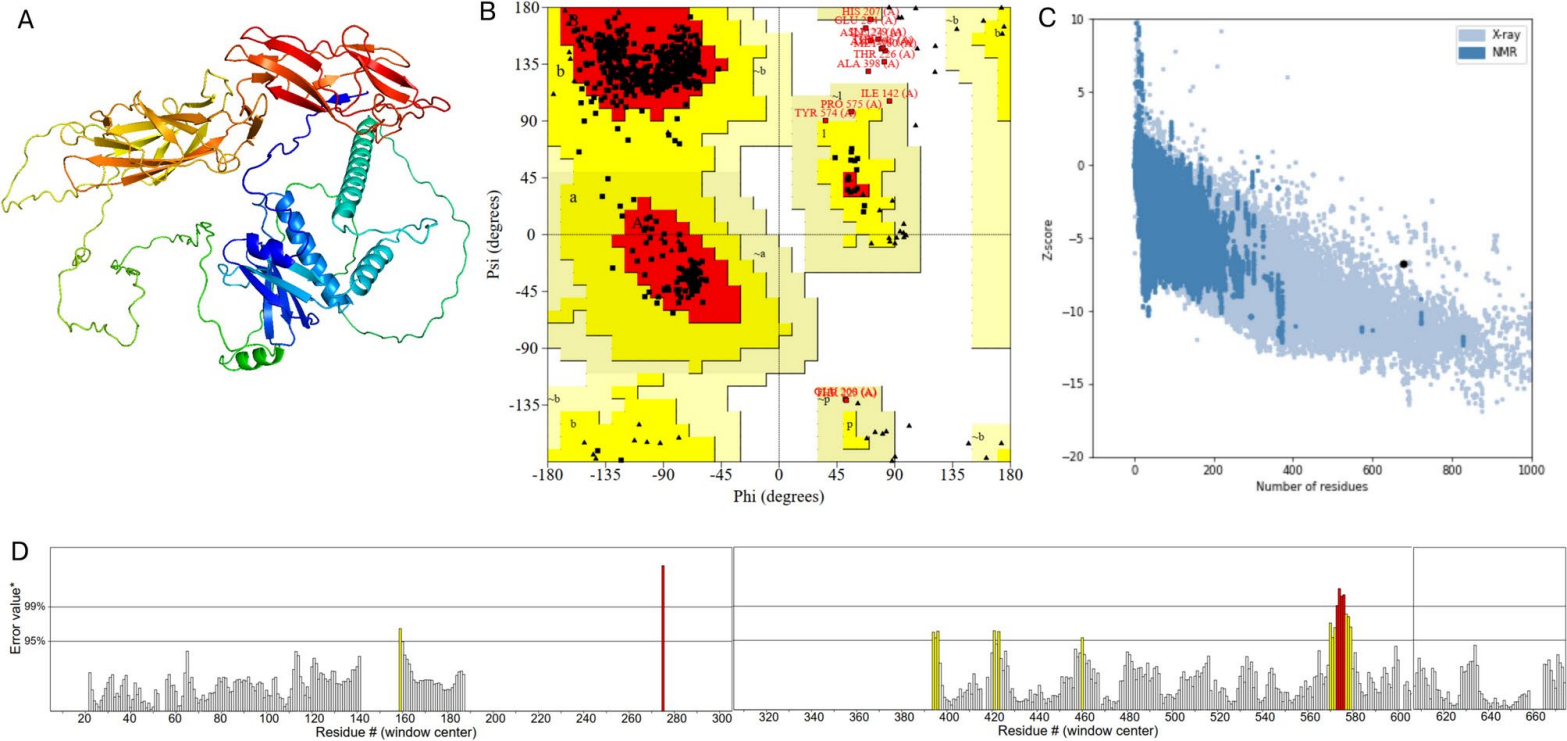
Three dimentional structure of the vaccine candidate. (**A**) Tertiary structure of refined Model 2 by GalaxyRefine, colored in rainbow from N-terminal (in blue) to C-terminal (in red). (**B**) Ramachandran plot by PROCHECK webserver. (**C**) Z-score calculated by Pro-SA webserver. (**D**) ERRAT program plot.

Table 4 summarizes the refined models, with model 5 emerging as the best, with a GDT-HA score of 0.8979 (the higher the value, the more accurate) and RMSD of 0.629 (a lower value indicates greater stability). This model’s 3D structure could be seen in a cartoon in Fig. 3A. To validate the quality of this 3D structure, the PROCHECK tool, ProSA-web, and ERRAT program were used. The Ramachandran plot revealed that 92.2%, 6.4%, and 1.4% of residues were found in the favored, allowed, and disallowed regions, respectively (Fig. 3B), with a Z-score of − 6.75 (Fig. 3C). Furthermore, this model had an ERRAT value of 95.65, which above 95% (Fig. 3D). According to the ERRAT algorithm, good high resolution structures generally yield results of 95% or above. Overall, model 5 (Fig. 3A) performed the best and was chosen as the vaccine candidate for further investigation.

### Discontinuous B-cell epitope prediction

**Fig. 4:**
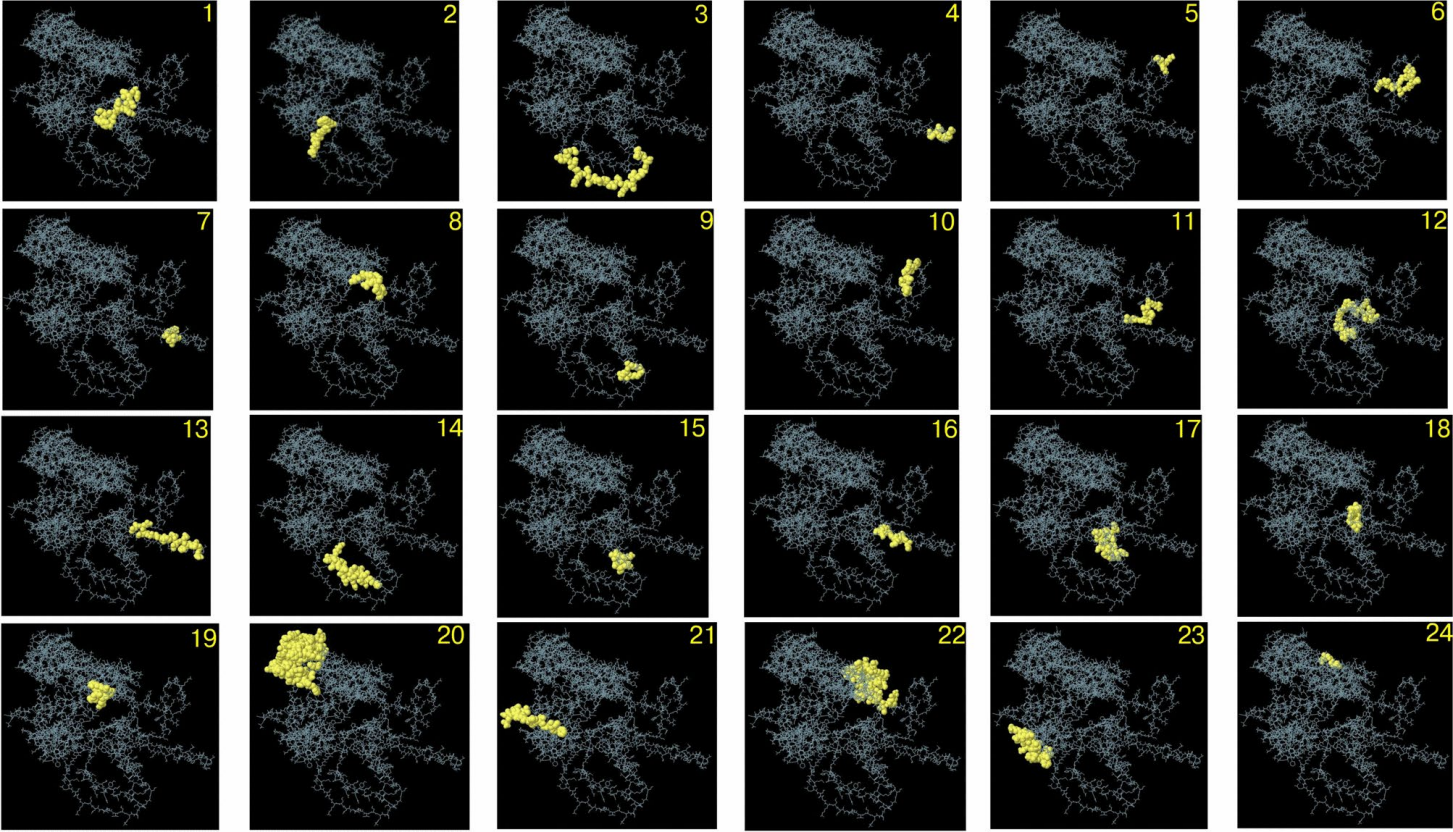
3D visualization of predicted discontinuous B-cell epitopes in the vaccine structure. Twenty-four discontinuous epitopes were identified, with their corresponding residue numbers and scores as follows: (**1**) 8 residues, score 0.898; (**2**) 7 residues, score 0.898; (**3**) 19 residues, score 0.898; (**4**) 6 residues, score 0.898; (**5**) 3 residues, score 0.898; (**6**) 12 residues, score 0.894; (**7**) 3 residues, score 0.890; (**8**) 8 residues, score 0.878; 14 4 residues, score 0.857; (**10**) 9 residues, score 0.830; (**11**) 8 residues, score 0.823; (**12**) 17 residues, score 0.791; (**13**) 1 residue, score 0.767; (**14**) 21 residues, score 0.742; (**15**) 4 residues, score 0.723; (**16**) 7 residues, score 0.723; (**17**) 14 residues, score 0.705; (**18**) 6 residues, score 0.649; 14 6 residues, score 0.633; (**20**) 91 residues, score 0.630; (**21**) 14 residues, score 0.630; (**22**) 35 residues, score 0.622; (**23**) 22 residues, score 0.595; and (**24**) 4 residues, score 0.516.

The discontinuous B-cell epitopes of the vaccine were predicted using the ElliPro tool. A total of 24 discontinuous epitopes were identified on the 3D structure of the vaccine, with protrusion scores ranging from 0.516 to 0.898. The size of the epitopes varied, comprising between 3 and 91 residues. The largest epitope, consisting of 91 residues, achieved a score of 0.630. These epitopes represent highly accessible regions on the vaccine surface, highlighting their potential to induce strong antibody responses. Detailed information about the predicted discontinuous epitopes is provided in Table 5, while their 3D structural representation is visualized in Fig. 4.

**Table 5:** Predicted discontinuous B-cell epitopes.

No	Residues	Number of residues	Score
1	A:N197, A:I198, A:S199, A:E200, A:N201, A:D202, A:D203, A:Y204	8	0.898
2	A:K206, A:H207, A:G208, A:G209, A:S210, A:Q211, A:Q212	7	0.898
3	A:M213, A:E214, A:S215, A:G216, A:K217, A:S218, A:L219, A:A220, A:Q221, A:T222, A:S223, A:K224, A:T225, A:T226, A:G227, A:G228, A:S229, A:G230, A:K231	19	0.898
4	A:V310, A:G311, A:G312, A:S313, A:L314, A:P315	6	0.898
5	A:D376, A:K377, A:L378	3	0.898
6	A:D379, A:E380, A:T381, A:L382, A:G383, A:G384, A:S385, A:K386, A:A387, A:K388, A:A389, A:K390	12	0.894
7	A:L317, A:I318, A:R319	3	0.890
8	A:S189, A:G190, A:G191, A:S192, A:E193, A:S194, A:L195, A:E196	8	0.878
9	A:K257, A:S258, A:R259, A:L260	4	0.857
10	A:V367, A:E368, A:V369, A:L370, A:G371, A:G372, A:S373, A:V374, A:K375	9	0.830
11	A:L358, A:G359, A:G360, A:S361, A:E362, A:Q363, A:A364, A:R365	8	0.823
12	A:C341, A:L342, A:L343, A:E344, A:T345, A:L346, A:G347, A:G348, A:S349, A:H350, A:P351, A:A352, A:C353, A:K354, A:L355, A:I356, A:R357	17	0.791
13	A:L296, A:E297, A:L298, A:G299, A:G300, A:S301, A:M302, A:V303, A:L304, A:E305, A:H306, A:P307, A:A308, A:R309	14	0.767
14	A:G262, A:G263, A:G264, A:S265, A:G266, A:L267, A:Y268, A:Q269, A:A270, A:L271, A:K272, A:E273, A:T274, A:H275, A:P276, A:H277, A:L278, A:I279, A:M280, A:G281, A:G282	21	0.742
15	A:V253, A:S254, A:N255, A:L256	4	0.723
16	A:L320, A:N321, A:M322, A:G323, A:G324, A:S325, A:Y326	7	0.723
17	A:L327, A:I328, A:P329, A:S330, A:D331, A:C332, A:S333, A:I334, A:G335, A:G336, A:S337, A:R338, A:P339, A:R340	14	0.705
18	A:E238, A:A239, A:R240, A:L241, A:A242, A:S243	6	0.649
19	A:G182, A:N184, A:S185, A:Q186, A:M187, A:R188	6	0.633
20	A:I2, A:V429, A:G430, A:G559, A:T570, A:L571, A:P572, A:D573, A:Y574, A:P575, A:G576, A:S577, A:V578, A:P579, A:I580, A:P581, A:L582, A:C586, A:A587, A:K588, A:S589, A:Q590, A:N591, A:L592, A:G593, A:Y594, A:T600, A:A601, A:D602, A:A603, A:G604, A:N605, A:S606, A:I607, A:F608, A:N610, A:F614, A:S615, A:P616, A:A617, A:Q618, A:G619, A:V620, A:G621, A:V622, A:Q623, A:L624, A:T625, A:R626, A:N627, A:G628, A:T629, A:I630, A:I631, A:P632, A:A633, A:N634, A:N635, A:T636, A:V637, A:S638, A:L639, A:G640, A:A641, A:V642, A:G643, A:T644, A:S645, A:A646, A:V647, A:S648, A:L649, A:G650, A:L651, A:T652, A:A653, A:N654, A:Y655, A:A656, A:R657, A:T658, A:G659, A:G660, A:Q661, A:V662, A:T663, A:A664, A:G665, A:N666, A:S669, A:Q678	91	0.630
21	A:F141, A:I142, A:G143, A:I144, A:P145, A:M146, A:G147, A:L148, A:P149, A:Q150, A:S151, A:I152, A:A153, A:L154	14	0.630
22	A:K391, A:Q393, A:I394, A:E395, A:A396, A:A397, A:A398, A:K399, A:N445, A:D446, A:Y447, A:P448, A:E449, A:T450, A:I451, A:T452, A:D453, A:Y454, A:R491, A:V492, A:V493, A:Y494, A:N495, A:S496, A:R497, A:T498, A:D499, A:K500, A:P501, A:N534, A:N535, A:Y536, A:N537, A:S538, A:D539	35	0.622
23	A:T40, A:L41, A:N42, A:D43, A:K44, A:F46, A:K64, A:N65, A:G66, A:A67, A:T99, A:E100, A:A101, A:K102, A:E104, A:A123, A:N124, A:E125, A:A126, A:K129, A:I130, A:Q133	22	0.595
24	A:S477, A:G478, A:S479, A:P503	4	0.516

This figure illustrates the 3D structure of the vaccine, highlighting the predicted discontinuous B-cell epitopes. These epitopes are represented as yellow spheres, indicating structurally accessible regions with immunogenic potential. The remaining residues of the vaccine are shown as gray sticks. The structural analysis underscores the potential of these epitopes to elicit robust immune responses, underscoring their critical role in the vaccine’s design as a multi-epitope candidate against lung cancer.

### Disulfide engineering of the vaccine

**Fig. 5:**
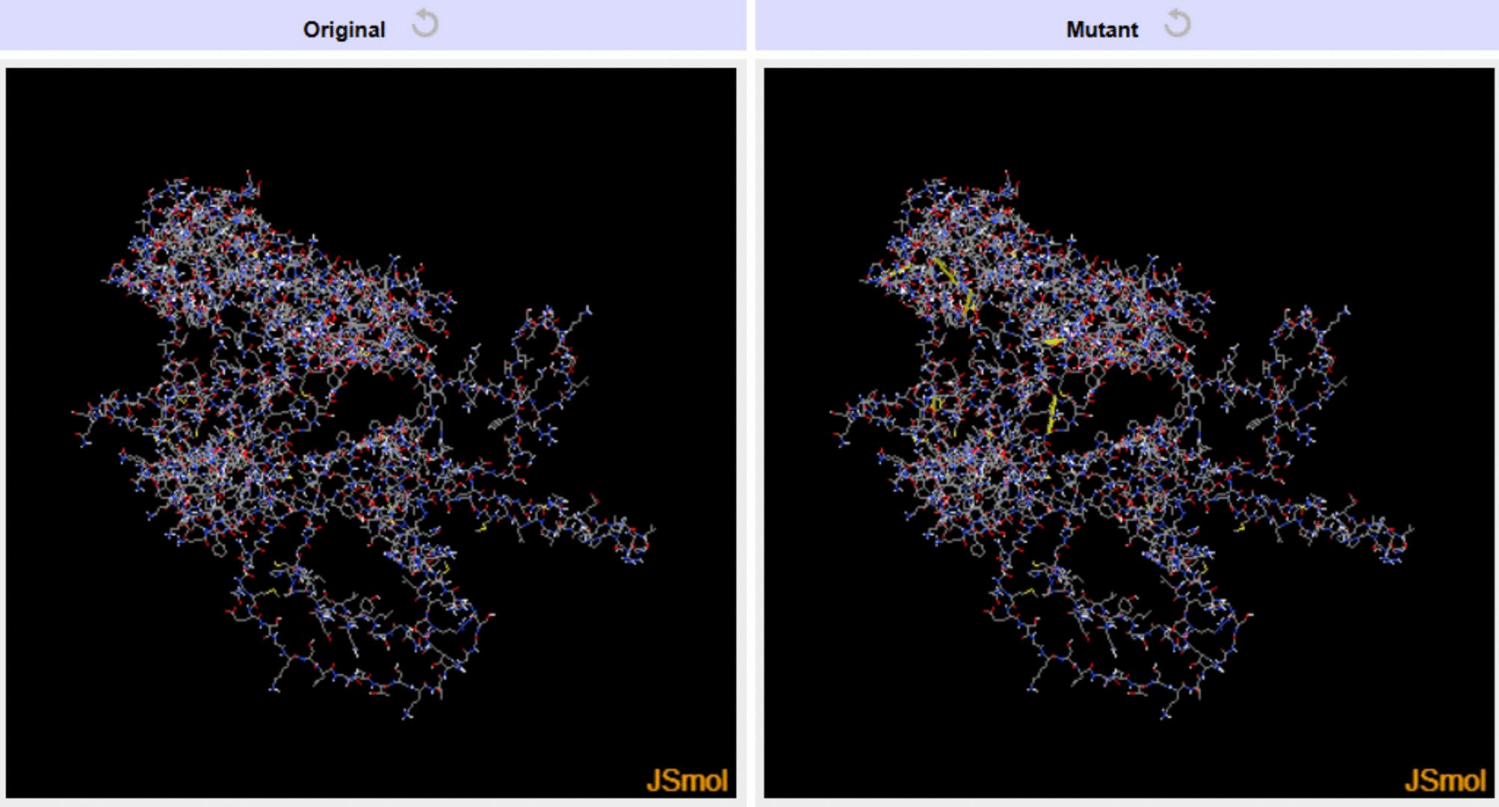
Disulfide engineering in the vaccine. (**A**) The original structure and (**B**) the mutant structure of the vaccine. In the mutant structure, the eight disulfide bonds are shown as yellow sticks.

Disulfide engineering was performed using the Disulfide by Design 2 v2.13 webserver to identify amino acid pairs capable of forming disulfide bonds. The analysis identified 43 candidate residue pairs with the potential for disulfide bond formation (Table S7). Among these, eight residue pairs were selected based on the feasibility of forming disulfide bonds (Table 6). Selection criteria included a χ^3^ angle ranging from − 87° to + 97° and an energy value below 2.2 kcal/mol. These residue pairs were predicted to form stable disulfide bonds, which can enhance the structural stability of the vaccine (Fig. 5).

**Table 6:** List of vaccine residue pairs with the feasibility of disulfide bond formation, along with their χ^3^ angle and energy scores.

No	Residue 1	Residue 2	χ^3^ angle (°)	Energy (kcal/mol)
1	23 Pro	28 Asp	78.34	1.37
2	23 Pro	29 Leu	− 97.31	1.00
3	74 Pro	78 His	99.54	1.34
4	464 Gly	524 Leu	− 95.31	0.47
5	602 Asp	607 Ile	107.47	2.13
6	612 Ala	666 Asn	− 97.19	0.68
7	612 Ala	668 Gln	109.07	2.07
8	622 Val	669 Ser	101.10	1.35

These selected residue pairs were subjected to mutation to cysteine, facilitating the formation of disulfide bonds. This engineering approach aims to enhance the structural stability of the vaccine construct, providing additional robustness for its functionality in various biological conditions.

### Molecular dynamics simulation studies

**Fig. 6:**
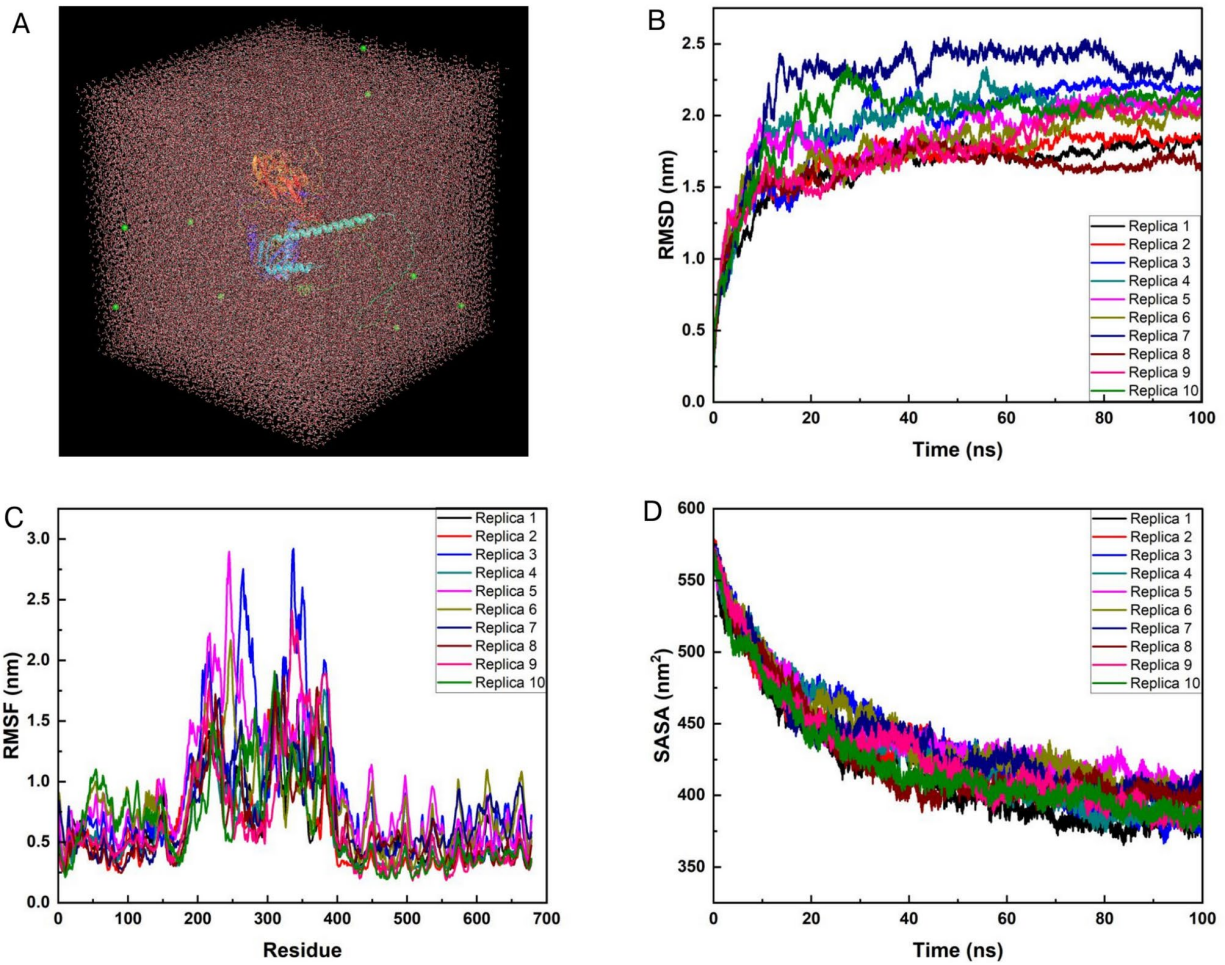
Molecular dynamics simulation of the vaccine candidate. (**A**) Solvation and ionization of the vaccine’s structure. 100,561 water molecules surround the system, and electrostatic neutrality was achieved by adding 10 chloride ions (represented as green spheres). (**B**–**D**) Root mean square deviation (RMSD), root mean square fluctuation (RMSF), and solvent-accessible surface area (SASA) of vaccine structure, respectively.

All-atom molecular dynamics (MD) simulations were conducted in ten replicates using GROMACS 2023 to evaluate the stability and dynamic behavior of the vaccine structure. The system was placed in an 8 × 8 × 8 nm cubic box, solvated with 100,561 water molecules, and neutralized with 10 chloride ions (Fig. 6A). The arrangement of water molecules and ions around the vaccine structure ensured proper system stabilization for subsequent molecular dynamics simulations. Key structural and dynamic properties such as root mean square deviation (RMSD), root mean square fluctuation (RMSF), and solvent-accessible surface area (SASA) were analyzed to evaluate the behavior of the vaccine candidate over the course of the 100 ns simulation.

The RMSD of the vaccine’s backbone residues was calculated to assess its structural stability during the simulation (Fig. 6B). After an initial equilibration period (~ 20 ns), the RMSD values stabilized at an average of 1.85 ± 0.19 nm across all replicates. This consistency across trajectories highlights the vaccine’s ability to maintain its overall structural integrity with only minor deviations. The stable RMSD values demonstrate that the vaccine structure did not undergo significant conformational changes during the simulation, supporting its structural robustness in a dynamic environment.

Additionally, the RMSF plot highlighted the flexibility of individual residues within the vaccine candidate (Fig. 6C). Higher fluctuations were observed in regions corresponding to loops and exposed surface residues, while lower fluctuations characterized the more rigid core regions of the protein. Notably, residues within the range of 250–400 exhibited the highest RMSF values, peaking between 2.0 and 3.0 nm, suggesting dynamic flexibility in these regions, likely corresponding to loops or surface-exposed domains. In contrast, residues below 100 and those between 450 and 678 displayed much lower RMSF values, typically around 0.5 nm or less, indicating that these regions were part of the protein’s more rigid core. The regions between residues 100–250 and 400–450 exhibited intermediate flexibility, reflecting a transition between the highly flexible and structurally constrained parts of the vaccine candidate. This distribution of flexibility aligns with the functional roles of flexible surface regions in protein–protein interactions, as they often play key roles in receptor binding and immune activation.

Furthermore, the SASA analysis provided a measure of the solvent exposure of the vaccine structure during the simulation (Fig. 6D). The SASA values initially decreased sharply, indicating slight structural compaction in the early phase of the simulation. After equilibration, the SASA values stabilized around an average of 434.22 ± 12.89 nm^2^, demonstrating consistent solvent exposure. This stability suggests that the vaccine candidate retained its overall structure without significant unfolding or further compaction during the simulation.

The results of the molecular dynamics simulations collectively confirm the structural stability and functional integrity of the vaccine candidate. The vaccine demonstrated stable backbone deviations (RMSD), moderate and biologically relevant residue flexibility (RMSF), and consistent solvent exposure (SASA) across all ten simulation replicates. These findings underscore the vaccine’s suitability for maintaining its structural and dynamic properties in a biologically relevant environment, supporting its potential efficacy as a therapeutic candidate.

### Molecular docking studies between vaccine and immune receptors

**Fig. 7:**
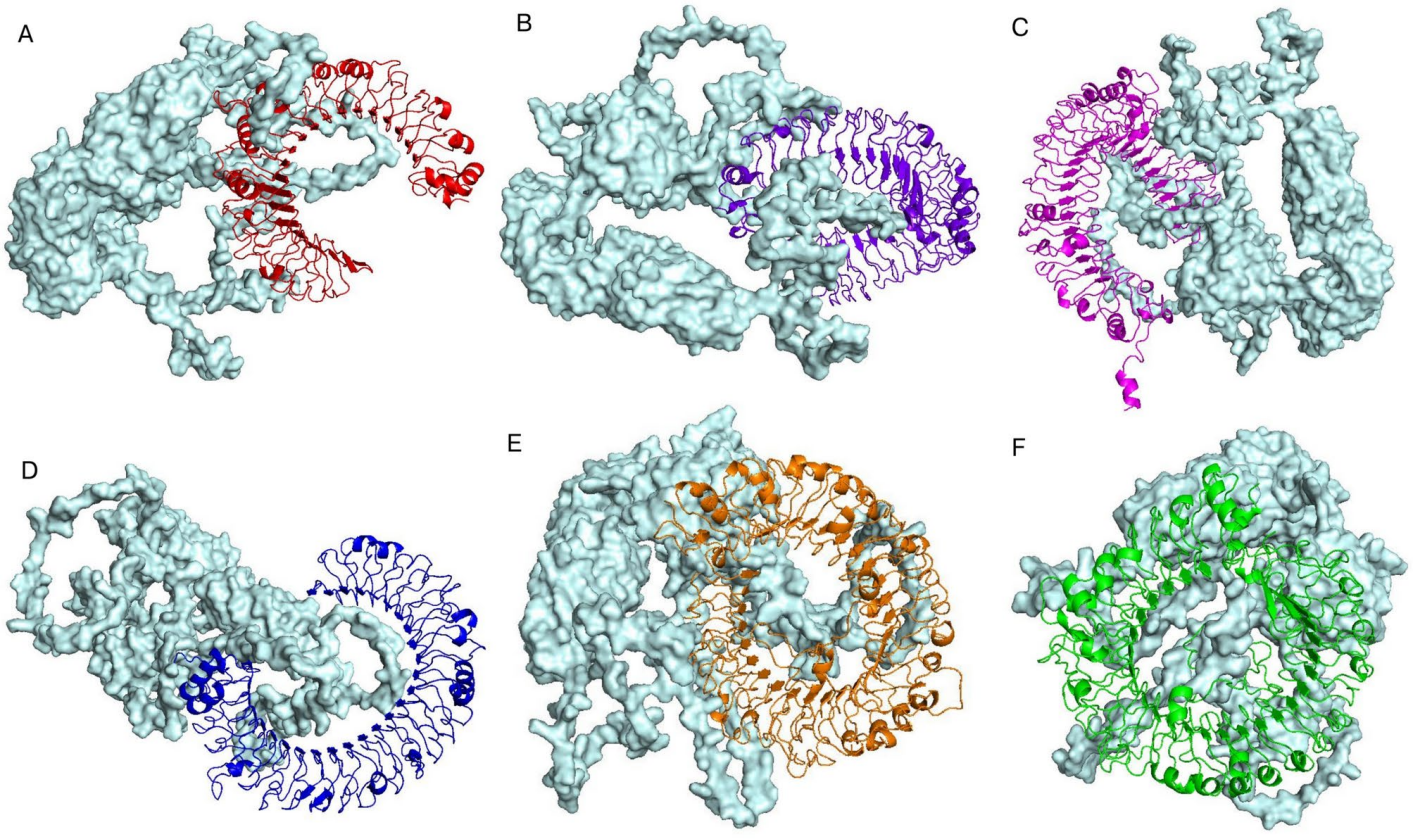
The docked complex of vaccine and TLRs. (**A**) Vaccine-TLR2 docked complex. (**B**) Vaccine-TLR4 docked complex. (**C**) Vaccine-TLR5 docked complex. (**D**) Vaccine-TLR3 docked complex. (**E**) Vaccine-TLR7 docked complex. (**F**) Vaccine-TLR8 docked complex. The vaccine is represented by the surface model, while TLRs are represented by the cartoon model.

Molecular docking studies were conducted to evaluate the interactions between the vaccine candidate and six selected TLRs: TLR2, TLR4, TLR5, TLR3, TLR7, and TLR8. The ClusPro v2.0 server was employed for docking, and the results were ranked based on cluster size, weighted score, and lowest energy score, representing the stability and strength of the interactions (Table 7 and Tables S8–S13). The best docking model for each vaccine-TLR complex is depicted in Fig. 7.

**Table 7:** Molecular docking of the vaccine with immune receptors.

Receptor	UniProt ID	Cluster size	Weighted score (kcal/mol)	Lowest energy (kcal/mol)	No. of hydrogen bonds	No. of salt bridges
TLR2 (26–579 aa)	O60603	40	− 1309.7	− 1475.0	42	6
TLR4 (30–624 aa)	O00206	44	− 1362.4	− 1468.3	35	3
TLR5 (21–639 aa)	O60602	66	− 1302.7	− 1916.8	47	1
TLR3 (25–696 aa)	O15455	51	− 1076.1	− 1364.1	25	2
TLR7 (28–831 aa)	Q9NYK1	31	− 1506.7	− 1663.4	38	6
TLR8 (31–818 aa)	Q9NR97	54	− 1393.8	− 1431.7	52	2

**Fig. 8:**
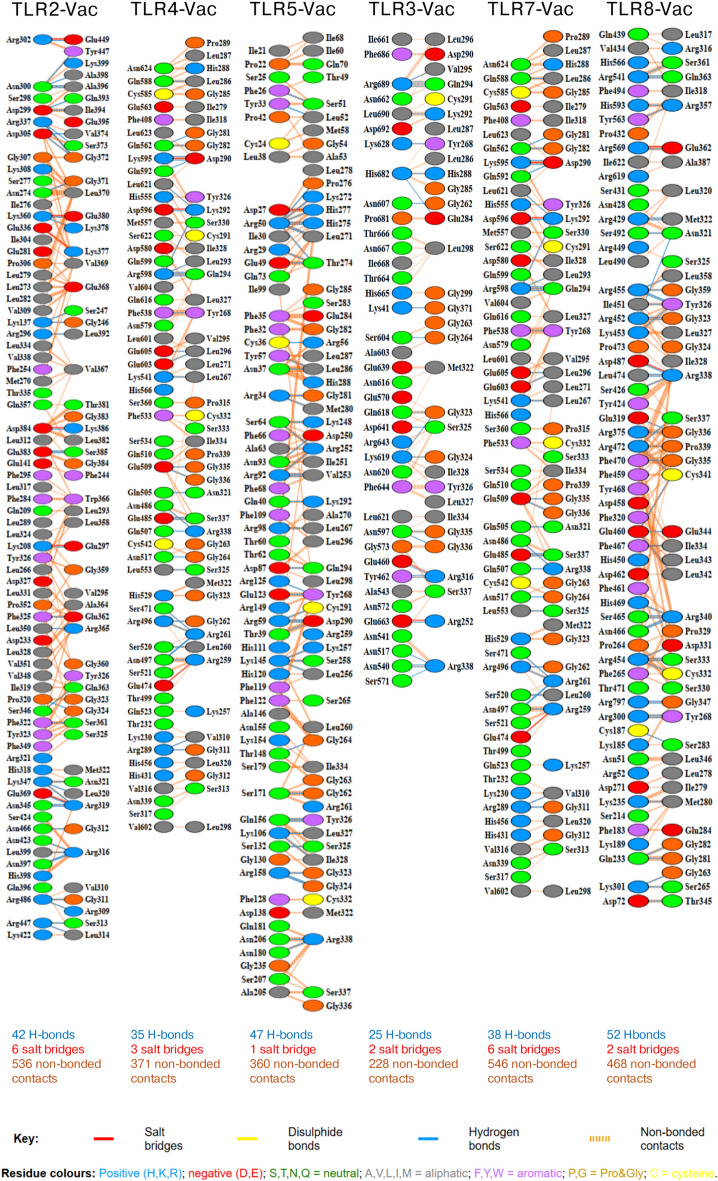
Residue interactions across TLRs-Vaccine interface. Coloured by residue type.

Furthermore, the number of hydrogen bonds and salt bridges formed within the docked complexes was analyzed using the PDBsum server to provide additional insights into the binding interactions. The residue interactions across the vaccine-TLR interfaces are presented in Fig. 8, while the lists of atom–atom interactions are detailed in Tables S14–S19.

Among the receptors, TLR5 exhibited the largest cluster size (66 members) and the most favorable interaction energy with a lowest energy score of − 1916.8 kcal/mol. TLR8 showed the highest number of hydrogen bonds and salt bridges (54), indicating strong molecular interactions. These results suggest that the vaccine forms stable interactions with all selected receptors, highlighting its potential to engage immune pathways effectively.

### Immune responses induced by the vaccine candidate

**Fig. 9:**
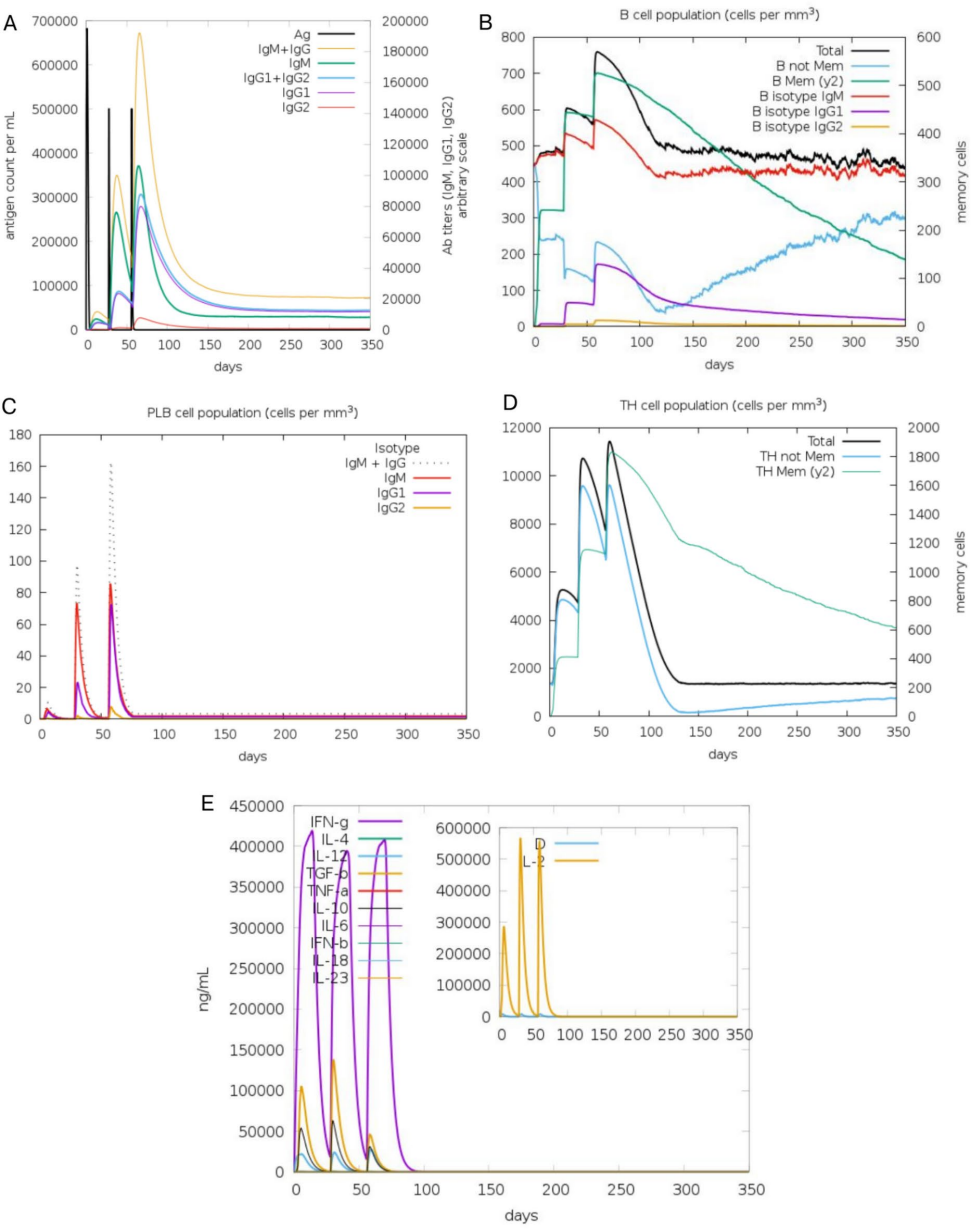
Immune simulation of the vaccine candidate. (**A**) Antigen count and antibody titers over time for IgM, IgG1, IgG2, and combined IgM + IgG responses following vaccine doses. (**B**) Dynamics of total B-cell and memory B-cell populations, including isotype-specific B-cells (IgM, IgG1, IgG2). (**C**) Plasma B-cell (PLB) populations producing antibodies, categorized by isotype. (**D**) T-helper (TH) and memory T-helper (TH Mem) cell populations, illustrating cellular immune activation. (**E**) Cytokine and interleukin concentrations, including IFN-γ, TGF-β, IL-10, and IL-12. The inset shows the danger signal curve, which reflects initial immune activation.

The immune response simulations were conducted to evaluate the immunogenic potential of the vaccine candidate, focusing on antigen count (Ag) along with antibody titers with specific subclasses, B-cell and T-cell dynamics, levels of interleukins and cytokines. As depicted in Fig. 9A, the Ag decreased sharply after each vaccine dose, indicating effective immune recognition and clearance. This was accompanied by a substantial increase in antibody titers, particularly IgG1 and IgM, demonstrating the vaccine’s ability to elicit robust humoral responses. The IgM response peaked shortly after the first dose, reflecting an early immune activation phase, followed by a consistent rise in IgG1 titers after subsequent doses. The IgG2 subclass showed a delayed but sustained increase, indicative of a long-term immune response, contributing to the vaccine’s immunological memory potential.

The B-cell population dynamics are shown in Fig. 9B. After each vaccine dose, there was a notable expansion in both naive and memory B-cell populations. Specifically, memory B-cells (labeled as “B Mem”) increased significantly, particularly after the second and third doses, suggesting the development of long-term immune memory. Subtype-specific B-cells responsible for IgG1 and IgM secretion were prominently active, aligning with the observed antibody titers.

Besides, plasma B-cell populations, which are responsible for antibody production, are presented in Fig. 9C. The vaccine induced strong plasma B-cell responses after the second and third doses. Plasma B-cell activity correlated with the rise in IgM and IgG1 titers, emphasizing their role in maintaining antibody levels.

T-helper cells (TH) and memory T-helper cells (TH Mem) play critical roles in orchestrating cellular immune responses, as shown in Fig. 9D. After each vaccine dose, there was a progressive increase in the total T-helper cell population, with a pronounced expansion of memory T-helper cells. The development of memory T-helper cells highlights the vaccine’s capacity to establish a robust and long-lasting cellular immune response, essential for anti-tumor immunity and pathogen clearance.

Furthermore, the vaccine candidate’s ability to stimulate cytokine production was analyzed to assess its immunomodulatory effects (Fig. 9E). The production of key cytokines such as IFN-γ (Interferon gamma) peaked early after each dose, indicating a strong Th1-biased immune response critical for antiviral and anti-tumor activity. Other cytokines, including TGF-β (Transforming Growth Factor Beta), IL-10, and IL-12, were produced in significant amounts, reflecting the activation of regulatory and effector T-cells. The inset plot highlights the early activation of danger signals, essential for initiating and amplifying the immune response.

The immune simulation results highlight the vaccine’s ability to generate strong humoral and cellular immune responses. The rapid antigen clearance, substantial increases in IgG1 and IgM titers, robust B-cell and T-helper cell populations, and significant cytokine production collectively underscore the vaccine’s high immunogenicity and potential to provide long-term immune protection. These findings demonstrate that the vaccine candidate effectively engages both innate and adaptive immune systems, supporting its potential as a therapeutic agent.

### Codon optimization and in silico cloning of the vaccine candidate

**Fig. 10:**
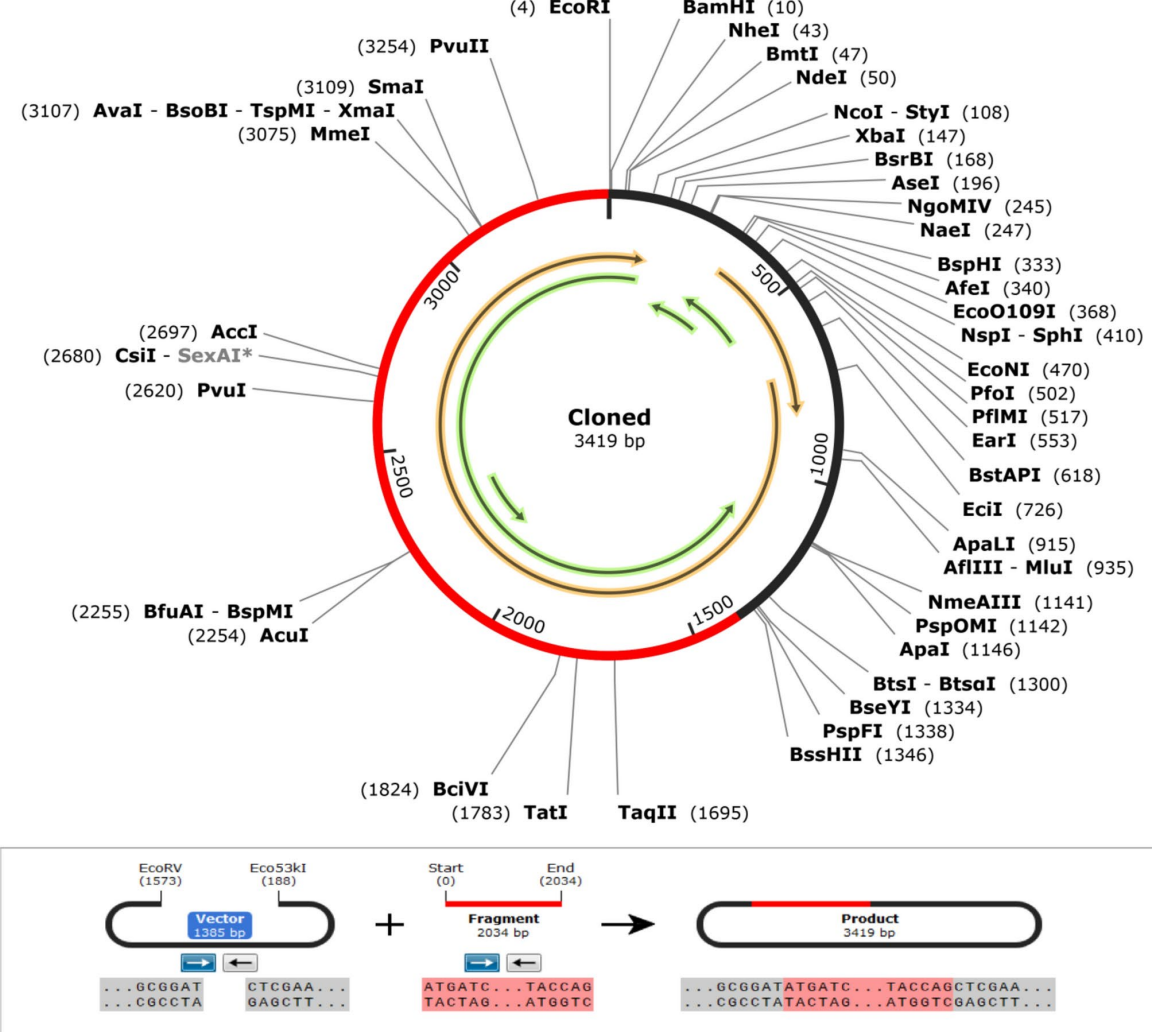
In silico cloning of the vaccine construct into the pET28a(+) vector.

Codon optimization using the JCat tool yielded a Codon Adaptation Index (CAI) of 1.0, indicating excellent adaptation of the vaccine construct to the *E. coli* K12 expression system. Additionally, the GC content of the codon-optimized sequence was 50.49%, which falls within the optimal range for *E. coli* expression. The in silico cloning of the vaccine construct into the pET28a( +) vector was successful, with the construct positioned between the EcoRV (1573) and Eco53kI (188) restriction sites (Fig. 10). The circular map illustrates the successful integration of the vaccine construct, with the cloned sequence spanning 3419 base pairs. The red segment represents the vaccine insert, while the black segment corresponds to the pET28a( +) backbone. Key restriction sites and their positions are annotated around the map for reference. Green arrows indicate the orientation of the vaccine gene transcription. Below the map, a schematic representation of the restriction cloning strategy is shown, including (i) the linearized vector, (ii) the vaccine fragment with cohesive ends, and (iii) the final ligation product containing the vaccine gene. The in silico cloned construct is now ready for downstream experimental validation.

## Discussion

Lung cancer, characterized by uncontrolled cell growth in lung tissues, remains one of the most lethal cancers globally. Primary lung cancers are predominantly carcinomas derived from epithelial cells and are categorized into small cell lung carcinoma (SCLC) and non-small cell lung carcinoma (NSCLC). While SCLC constitutes only 15% of cases, NSCLC represents 85% and includes large cell carcinoma, squamous cell carcinoma, and adenocarcinoma^14^. Lung cancer is one of the leading causes of death globally in both females and males^15^. In the United States, it claims more lives each year than breast, prostate, pancreatic, and Non-Hodgkin lymphoma combined^2^. Given its high incidence and mortality rates worldwide, effective prevention and treatment strategies are urgently needed.

Vaccines supported by immunoinformatics represent a promising strategy in combating lung cancer, offering the potential to transform cancer therapy in the near future. This study aimed to develop a therapeutic lung cancer vaccine targeting tumor pyroptosis-associated antigens (CARD8, NAIP, NLRP1, and NLRP3) using immunoinformatics approaches. The selection of these proteins as candidate tumor-associated antigens for vaccine development was guided by their significant roles in apoptosis, immune activation, and tumor immunology. Data from the Human Protein Atlas Database confirmed that these proteins are predominantly expressed in the cytoplasm across various tissues, ensuring their accessibility as antigens. Notably, in lung adenocarcinoma, high expression levels of these proteins have been associated with improved overall survival (OS), further underscoring their relevance in cancer immunotherapy^13^. CARD8 has been shown to induce pyroptosis, a programmed cell death mechanism, in leukemic cells, making it a promising target for cancer vaccines^16^. NLRP1 has exhibited tumor-suppressive effects in colon cancer and is also implicated in innate immunity, emphasizing its dual role in both tumor control and immune activation^17^. Furthermore, NLRP1, NLRP3 and NAIP are members of the NOD-like receptor (NLR) family, which plays critical roles in both innate immune responses and tumorigenesis^16^. The ability of these NLR proteins to mediate tumor development and immune regulation underscores their potential to elicit robust anti-tumor immune responses. Zhou et al. further demonstrated a strong correlation between these proteins and antigen-presenting cells (APCs), suggesting that antigens derived from these proteins are effectively processed and presented to T cells^13^. This is a critical process for initiating adaptive immune responses against tumor cells. By contributing to apoptosis and pyroptosis, these proteins may also promote tumor cell destruction through cell death pathways. Collectively, these findings establish CARD8, NAIP, NLRP1, and NLRP3 as robust tumor-associated antigens, justifying their selection for vaccine development. Their involvement in apoptosis, immune activation, and antigen presentation underscores their potential to mediate a specific and potent anti-tumor immune response.

Previous studies have successfully employed immunoinformatics for vaccine design against various cancers and pathogens^18–23^. In previous studies, epitopes of MAGE-A3, EGF, and MUC-1 proteins^24^, G protein-coupled receptor 56 (GPR56)^25^, alpha-enolase, vimentin, and carcinoembryonic antigen fragment^26^, G-protein coupled receptor 87 (GPCR-87), cystine/glutamate transporter (SLC7A11), Immunoglobulin binding protein 1 (IGBP1), and thioredoxin domain-containing protein 5 (TXNDC5)^27^ have been targeted for lung cancer vaccines development using immunoinformatics. In this study, we identified T-cell and B-cell epitopes from the tumor pyroptosis-associated antigens and evaluated their toxicity, allergenicity, antigenicity, and capacity to induce IFN-γ and interleukin production. Epitopes with VaxiJen scores above 1.1, which were non-toxic, non-allergenic, and capable of inducing IFN-γ and interleukins, were selected for vaccine development. The selected T-cell epitopes are predicted to provide global population coverage of 77.21%. The final vaccine design incorporated these epitopes along with cholera toxin subunit B, *E. coli* FimH, and TpD adjuvants, linked by GGS and EAAAK linkers. Previous studies have demonstrated the effectiveness of TpD in protecting mucosal surfaces and promoting neutralizing antibody production^28,29^, while cholera toxin subunit B^30^ and *E. coli* FimH have shown potential to enhance immunogenicity and stimulate cytokine production^31,32^. Linkers were incorporated to ensure the proper assembly and flexibility and to maintain the structural integrity of the multi-epitope vaccine. The EAAAK linkers were used to connect the adjuvants due to their rigidity, which helps maintain structural separation and functional independence of the fused domains^33^. The GGS linkers were used to connect epitopes because of their flexibility, which improves folding, accessibility, and proteasomal cleavage, facilitating effective antigen presentation^33,34^.

The designed vaccine exhibited favorable physicochemical and immunological properties, making it a strong candidate for further development. Its stability index (35.98) and high antigenicity (VaxiJen: 0.6472) suggest robust structural integrity and strong immunogenic potential. The vaccine was predicted to be non-allergenic and non-toxic, ensuring safety in therapeutic applications. The solubility score (0.904) obtained from SoluProt v1.0 highlights the vaccine’s high solubility, indicating its suitability for efficient expression in *E. coli*. Other properties, such as its thermostability (aliphatic index: 85.60) and hydrophilic nature (GRAVY: − 0.162), further support its suitability for storage, transport, and interaction with biological systems. These findings underscore the vaccine’s potential for robust immune activation, safety, and efficient production, paving the way for experimental validation and large-scale application.

The 3D structure of vaccine candidate was modeled, refined, and validated, followed by discontinuous B-cell epitope and disulfide engineering prediction. Using the ElliPro tool, twenty-four epitopes were identified with scores ranging from 0.516 to 0.898. These epitopes were represented as yellow spheres within the 3D model, while the remaining residues of the vaccine structure were depicted as gray sticks. These results underscore the distribution and structural context of epitopes, which are critical for assessing the antigenic potential of the vaccine candidate. Disulfide engineering of the vaccine was performed using the Disulfide by Design 2 (DbD2) v2.13 webserver. A total of 43 candidate residue pairs were identified as capable of forming disulfide bonds based on their χ^3^ angles and energy values. From these, eight residue pairs were selected due to their favorable χ^3^ angles (ranging from − 87° to + 97°) and energy values below 2.2 kcal/mol, indicating feasibility for stable disulfide bond formation. These selected residue pairs were mutated to cysteine, facilitating the formation of disulfide bonds. This finding highlights the enhanced structural stability of the vaccine construct, as these engineered disulfide bonds provide additional robustness for functionality under various biological conditions.

The molecular dynamics simulation provided a comprehensive evaluation of the vaccine’s structural integrity and dynamic properties. The consistent RMSD trajectories across 10 replicates indicate that the vaccine candidate achieved structural equilibrium after 30 ns, underscoring its stability in a solvated environment. RMSF analysis further elucidated the dynamic behavior of individual residues, with higher flexibility observed in loop regions, which is characteristic of vaccine structures. The reduction in SASA values over the simulation duration reflects the vaccine’s compactness and minimized exposure of hydrophobic regions, contributing to its stability. These findings align with previous studies that emphasize the importance of compact and stable vaccine structures in eliciting robust immune responses^35–37^. The molecular dynamics simulation results thus validate the structural reliability and flexibility of the vaccine, strengthening its potential as an effective immunotherapeutic candidate.

TLRs play a crucial role in recognizing pathogen-associated molecular patterns (PAMPs) and activating immune responses, which are essential for an effective vaccine response^38^. Molecular docking studies with TLRs in this study reveal the vaccine candidate’s potential to elicit strong immune responses by effectively interacting with key immune receptors. The docking analysis demonstrated stable interactions between the vaccine and the extracellular domains of six selected TLRs, underscoring its potential to activate immune pathways involved in tumor immunology, pyroptosis, and immune regulation. Among the receptors, TLR5 exhibited the strongest binding based on cluster size (66 members) and the lowest energy score (− 1916.8 kcal/mol), indicating its significant role in vaccine-receptor interactions. These findings align with prior studies highlighting TLR5’s critical role in immune activation. TLR5 recognizes bacterial flagellin, a conserved component of bacterial motility, which structurally mimics the motifs in epitope-based vaccines^39–41^. Flagellin recognition by TLR5 bridges innate and adaptive immunity, enhancing vaccine immunogenicity through targeted activation of TLR5-mediated pathways^39^. Furthermore, the mimicry of flagellin by epitope-based vaccines has been emphasized in recent research as a promising approach to boost immune responses^40,41^. TLR7 and TLR8, members of the endosomal TLR family, also exhibited substantial binding strength. TLR8, in particular, formed the highest number of hydrogen bonds (52), suggesting robust interactions primarily mediated by electrostatic forces. These receptors are essential for recognizing single-stranded RNA and are known to play significant roles in antiviral immunity and cancer immunology. The balanced interaction energies observed across all tested receptors underline the vaccine candidate’s ability to comprehensively engage innate immune mechanisms. The inclusion of multiple TLRs broadens the scope of immune activation, potentially generating synergistic effects that enhance anti-tumor responses. Collectively, these findings support the vaccine candidate’s potential as a vaccine capable of stimulating both innate and adaptive immunity through effective engagement with TLRs.

Additionally, the immune simulation results indicated that the lung cancer vaccine candidate effectively induced a robust immune response. Following each vaccine dose, the progressive increase in antibody titers, particularly IgG1 and IgM, suggested strong antigen recognition and a durable humoral response. The expansion of B-memory cells and plasma B cells further highlighted the vaccine’s capacity to elicit long-term immunity. Furthermore, the observed rise in T-helper and memory T-helper cell populations reflected the activation of a potent cellular immune response. The production of cytokines, such as IL-10, IL-12, and IFN-γ, underscored the vaccine’s ability to stimulate both adaptive and innate immune pathways, suggesting its potential efficacy in promoting a comprehensive immune defense against lung cancer.

Codon optimization is a critical step in ensuring efficient expression of recombinant proteins in bacterial hosts. The vaccine construct exhibited a CAI value of 1.0 and a GC content of 50.49%, both of which are within the optimal range for protein expression in *E. coli*^42,43^. A CAI value close to 1.0 reflects a high level of adaptation to the codon usage of the host organism, enhancing translational efficiency. Meanwhile, a balanced GC content, as observed in this study, minimizes the potential for secondary structure formation in mRNA, which can otherwise impede transcription and translation processes^42^. The in silico cloning process further demonstrated the feasibility of inserting the codon-optimized vaccine gene into the *E. coli* expression vector pET28a( +). Restriction sites EcoRV and Eco53kI were successfully used for the insertion, ensuring compatibility and ease of downstream applications. This approach confirms that the codon optimization strategy not only enhances expression potential but also facilitates efficient molecular cloning, which is vital for experimental validation and large-scale production. These results collectively underscore the robustness of the designed vaccine construct for bacterial expression systems. The integration of codon optimization and in silico cloning ensures that the vaccine candidate is primed for efficient expression, paving the way for its experimental validation and application in vaccine development pipelines.

## Conclusion

This study successfully demonstrates the potential of a multi-epitope vaccine designed against lung cancer by leveraging immunoinformatics and bioinformatics approaches. The selected antigens, CARD8, NAIP, NLRP1, and NLRP3, were validated as promising targets due to their roles in pyroptosis, immune activation, and correlation with improved survival in lung adenocarcinoma patients. The vaccine construct exhibited favorable antigenic and physicochemical properties, including solubility and stability, and its interaction with immune receptors indicated its ability to activate comprehensive immune responses. The robust structural stability of the vaccine, validated through molecular dynamics simulations, and the promising immune responses observed in simulations underscore its immunogenic potential. This work provides a foundation for future experimental validation and preclinical studies, advancing the development of effective immunotherapies for lung cancer.

## Methods and materials

### Sequence retrieval

The reviewed (Swiss-Prot) amino acid sequences of the tumor pyroptosis-associated antigens CARD8, NAIP, NLRP1, and NLRP3 were obtained from the UniProt database^44^.

### Prediction and analysis of T-cell and B-cell epitopes

T-cell and B-cell epitopes were predicted using several computational tools. For T-helper cell epitopes binding to MHC class II molecules, NetMHCIIpan-4.3 was utilized^45^. T-cytotoxic cell epitopes binding to MHC class I molecules were predicted using NetMHCpan-4.1^46^. Protein sequences were inputted in FASTA format, with peptide lengths of 9 and 15 chosen for NetMHCpan-4.1 and NetMHCIIpan-4.3, respectively. The default thresholds for strong and weak binders were used.

For B-cell epitope prediction, the BepiPred-3.0 linear epitope prediction method available on the IEDB website was employed^47^. The antigenicity of the predicted epitopes was assessed using VaxiJen v.2.0^48^. AllerTop v.2.0, ToxinPred, and IFNepitope servers were used to evaluate the allergenic potential, toxicity, and IFN-γ activation potential of the epitopes, respectively^49–51^. The IL4Pred and IL10Pred webservers were also employed to assess the ability of the epitopes to induce IL-4 and IL-10 production^52,53^.

### Population coverage analysis

To estimate population coverage for the selected T-cell epitopes in the vaccine candidate, the IEDB population coverage analysis tool was used^54^. Due to the lack of available tools, population coverage for B-cell epitopes was not calculated. In the IEDB tool, default values were used for the number of epitopes and query options, while the “World” option was selected for area/population. Both Class I and II epitopes were analyzed in combination.

### Designing the lung cancer vaccine candidate and analysis

The vaccine candidate was designed by selecting epitopes that met specific criteria: non-allergenic, non-toxic, antigenic, and capable of inducing IFN-γ production. Cholera toxin subunit B (CTB), epitopes from tetanus and diphtheria toxoids (TpD), and the final subunit of *Escherichia coli* type 1 fimbria (FimH) were used as adjuvants^55^. The adjuvants were connected with EAAAK linkers, while epitopes were linked together using GGS linkers.

The ProtParam web tool was used to evaluate the physicochemical properties of the designed vaccine^56^. To assess the solubility of the vaccine candidate, the SoluProt v1.0 server, an established platform for predicting protein solubility in *E. coli* expression systems, was employed^57^. The server computes a solubility score based on the physicochemical properties of the protein sequence, with scores above 0.5 indicating soluble expression and scores below 0.5 suggesting insoluble expression. To evaluate antigenicity, allergenicity, and toxicity, the vaccine candidate was analyzed using VaxiJen v2.0, AllerTop v2.0, and ToxinPred, respectively. Additionally, BLASTp analysis (taxid: 9606; *Homo sapiens*) was performed to identify homologous human proteins^58^.

### Structure prediction, refinement, and validation of the vaccine candidate

The secondary structure of the vaccine was predicted using PDBsum^59^, using Gail Hutchinson’s PROMOTIF v.3.0 program^60^. The 3D structure of the vaccine was predicted using three servers: SWISS-MODEL^61^, Iterative Threading ASSEmbly Refinement (I-TASSER)^62^, and AlphaFold2 via AlphaFold Colab^63^. To refine the 3D structure, the GalaxyRefine module of GalaxyWEB was employed^64^. Structure validation was performed using the PROCHECK v.3.5^65^, ProSA-web^66^, and ERRAT program^67^.

### Discontinuous B-cell epitope prediction

Discontinuous (conformational) B-cell epitopes were predicted using the ElliPro webserver^68^. ElliPro identifies discontinuous epitopes by integrating residue protrusion indices and spatial clustering on the surface of a 3D protein structure. For this analysis, the 3D structure of the vaccine construct was uploaded to ElliPro, and default parameters were used, including a protrusion index (PI) threshold of 0.5 and a maximum distance of 6 Å. Predicted epitopes were ranked based on their protrusion scores, which correlate with their accessibility and likelihood of recognition by antibodies.

### Disulfide engineering of the vaccine

To predict amino acids that can form disulfide bonds and improve the stability of the vaccine disulfide engineering was done using Disulfide by Design 2 v2.13 webserver^69^. The disulfide engineering of the vaccine was performed by using the default parameters of the web server. Then, the amino acid pairs with energy below 2.2 kcal/mol were mutated to cysteine to form disulfide bonds because about 90% of the native disulfide bonds have energy below 2.2 kcal/mol^70^.

### Molecular dynamics simulation studies

All-atom molecular dynamics (MD) simulations were carried out using GROMACS 2023 on a Linux platform to evaluate the stability and dynamic behavior of the 3D vaccine structure, employing the CHARMM27 force field^71^. The vaccine structure was placed in an 8 × 8x8 nm cubic simulation box and solvated with 100,561 water molecules. The system was neutralized by adding 10 chloride ions. Energy minimization was conducted using the steepest descent algorithm for 50,000 steps. This was followed by two equilibration stages: NVT (constant volume and temperature) at 300 K and NPT (constant pressure and temperature) at 1 bar, each for 50,000 steps (100 ps). The production simulation ran for 100 ns (50,000,000 steps) under NPT conditions, involving 311,885 atoms. Post-simulation analyses, including root mean square deviation (RMSD), root mean square fluctuation (RMSF), and solvent-accessible surface area (SASA), were conducted to evaluate structural stability and flexibility. To ensure accuracy, ten replicate simulations were performed, each with varying initial velocities, and all were run for 100 ns.

### Molecular docking studies between vaccine and immune receptors

Among the 10 human TLRs, TLR2, TLR4, TLR5, TLR3, TLR7, and TLR8 were selected for molecular docking studies due to their biological roles in pyroptosis, tumor immunology, and critical involvement in immune pathways essential for eliciting a robust anti-tumor response from the multi-epitope vaccine candidate^72–75^. These TLRs were chosen to ensure a balanced strategy for activating both innate and adaptive immunity, thereby maximizing the therapeutic potential of the vaccine candidate. The 3D structures of the selected TLRs were retrieved from the AlphaFold Protein Structure Database^76^ with corresponding UniProt IDs. For all six receptors, only the extracellular domains, encompassing the amino acid residues critical for ligand recognition and binding, were retained, while other regions were excluded to focus the docking studies.

Molecular docking studies were performed using the ClusPro v2.0 server, a widely used protein–protein docking server that performs rigid-body docking by sampling billions of conformations^77^. It utilizes the Fast Fourier Transform (FFT) correlation approach to evaluate interaction energies across a vast number of docked conformations on a grid. The server retains the top 1000 lowest-energy structures and employs root-mean-square deviation (RMSD)-based clustering to identify the largest clusters, which are considered the most probable models of the complex^77^. Subsequently, these selected structures undergo energy minimization refinement to optimize their stability and accuracy^77^. The top-ranked docking poses were further analyzed using PDBsum to identify key interactions, including hydrogen bonding, salt bridges, and list of atom–atom interactions across protein–protein interface, which are critical for stable binding between the vaccine and immune receptors^78^.

### Immune simulation analysis

To assess the immune response elicited by the vaccine construct, an in-silico immune simulation was performed using C-IMMSIM software^79^. Default parameters were applied, with adjustments made only to the time step settings. Following recommendations to maintain at least a four-week interval between vaccine dose^80,81^, three doses were simulated at 4-week intervals. Time steps of 1, 84 (approximately 4 weeks), and 168 (approximately 8 weeks) were used to simulate the immune response profile.

### Codon optimization and in silico cloning of the vaccine candidate

Codon optimization of the vaccine construct gene was performed using the JCat web tool to ensure compatibility with the expression system in the *E. coli* K12 strain and enhance translation efficiency^43^. This optimization maximized the Codon Adaptation Index (CAI) while maintaining the amino acid sequence integrity and avoiding codons associated with ribosomal stalling or low expression efficiency. The SnapGene restriction cloning module was then used for in silico cloning. The codon-optimized vaccine construct was inserted into the pET28a( +) vector between the EcoRV (1573) and Eco53kI (188) restriction sites to complete the cloning process.

## Supplementary Information

Supplementary Material 1.

Supplementary Material 2.

## Data Availability

All data generated or analyzed during this study are included in this published article (and its Supplementary Information files).
