# Supplementary material for: Identification and evaluation of tumor pyroptosis-associated antigens for design a vaccine candidate against lung cancer
Source: Sci Rep. 2026 Mar 19;16:9559. doi: 10.1038/s41598-024-84792-4 (PMC13009229; doi:10.1038/s41598-024-84792-4)
Supplement: Supplementary file 2 — Supplementary Material 2. [file 41598_2024_84792_MOESM2_ESM.pdf]

Supplementary Information for

Identification and evaluation of tumor pyroptosis-associated antigens for design a vaccine candidate against lung cancer

Truc Ly Nguyen[1], Heebal Kim[1,2,3*]

[1] Department of Agricultural Biotechnology and Research Institute of Agriculture and Life Sciences, Seoul National University, Seoul 08826, Republic of Korea

[2] Interdisciplinary Program in Bioinformatics, Seoul National University, Seoul 08826, Republic of Korea

[3] eGnome, Inc., Seoul 05836, Republic of Korea

*Corresponding author: Heebal Kim. heebal@snu.ac.kr

I. Supplementary Figure

Figure S1. The three-dimensional structure and information of model 1 predicted by SWISS-MODEL.

Figure S2. Prediction and validation of three-dimensional structure of the vaccine construct. (**A**) The 3D structure of rank_1 (model 1) predicted by I-TASSER representation in cartoon retrieved from PyMOL. (**B**) Ramachandran plot validation of model 1 by PROCHECK tool. (**C**) The 3D structure of model 3 after refining by GalaxyRefine representation in cartoon retrieved from PyMOL. (**D**) Ramachandran plot validation of refined structure by PROCHECK tool.

Figure S3. Prediction and validation of three-dimensional structure of the vaccine construct. (A) Predicted LDDT per residue for the 5 models obtained from AlphaFold Colab. (B) The 3D structure of rank_1 (model 2) representation in cartoon retrieved from PyMOL. (C) Ramachandran plot validation by PROCHECK tool.

II. Supplementary Table

Table S1. Sequence of the protein CARD8 (UniProt ID: Q9Y2G2).

Table S2. Sequence of the protein NAIP (UniProt ID: Q13075).

Table S3. Sequence of the protein NLRP1 (UniProt ID: Q9C000).

Table S4. Sequence of the protein NLRP3 (UniProt ID: Q96P20).

Table S5. Structure information of 5 models predicted by I-TASSER.

Table S6. Structure information of 5 refined models obtained from GalaxyRefine.

Table S7. Predicted disulfide bonds with corresponding residue pairs, angles, and energy values, using Disulfide by Design 2 v2.13 server.

Table S8. Docking result of vaccine with TLR2, generated by ClusPro v2.0 server.

Table S9. Docking result of vaccine with TLR4, generated by ClusPro v2.0 server.

Table S10. Docking result of vaccine with TLR5, generated by ClusPro v2.0 server.

Table S11. Docking result of vaccine with TLR3, generated by ClusPro v2.0 server.

Table S12. Docking result of vaccine with TLR7, generated by ClusPro v2.0 server.

Table S13. Docking result of vaccine with TLR8, generated by ClusPro v2.0 server.

Table S14. List of atom-atom interactions across vaccine-TLR2 interface.

Table S15. List of atom-atom interactions across vaccine-TLR4 interface.

Table S16. List of atom-atom interactions across vaccine-TLR5 interface.

Table S17. List of atom-atom interactions across vaccine-TLR3 interface.

Table S18. List of atom-atom interactions across vaccine-TLR7 interface.

Table S19. List of atom-atom interactions across vaccine-TLR8 interface.

I. Supplementary Figure

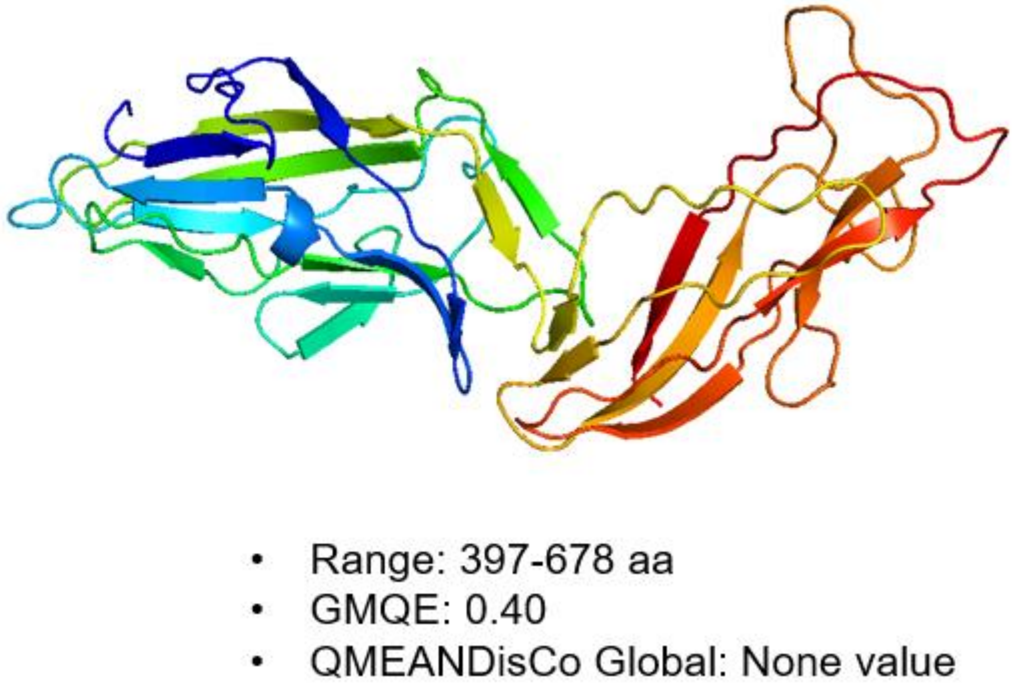

Figure S1. The three-dimensional structure and information of model 1 predicted by SWISS-MODEL.

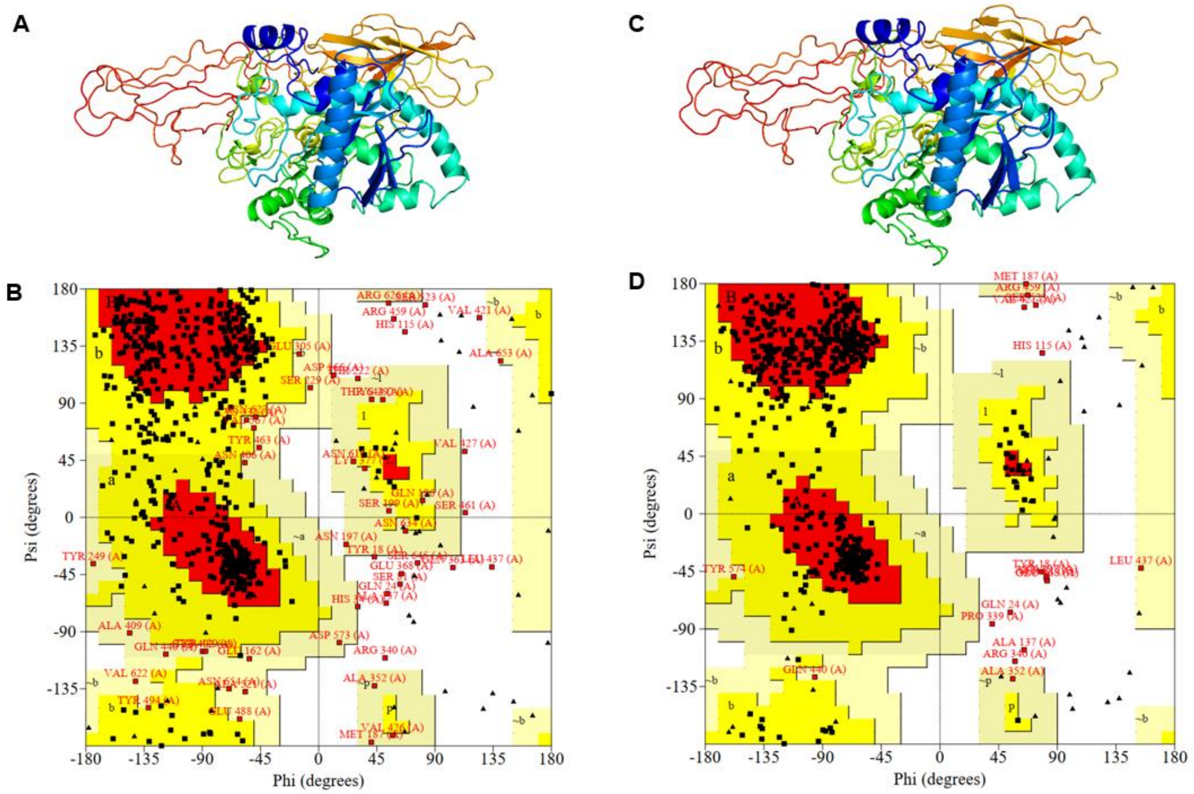

Figure S2. Prediction and validation of three-dimensional structure of the vaccine construct. (**A**) The 3D structure of rank_1 (model 1) predicted by I-TASSER representation in cartoon retrieved from PyMOL. (**B**) Ramachandran plot validation of model 1 by PROCHECK tool. (**C**) The 3D structure of model 3 after refining by GalaxyRefine representation in cartoon retrieved from PyMOL. (**D**) Ramachandran plot validation of refined structure by PROCHECK tool.

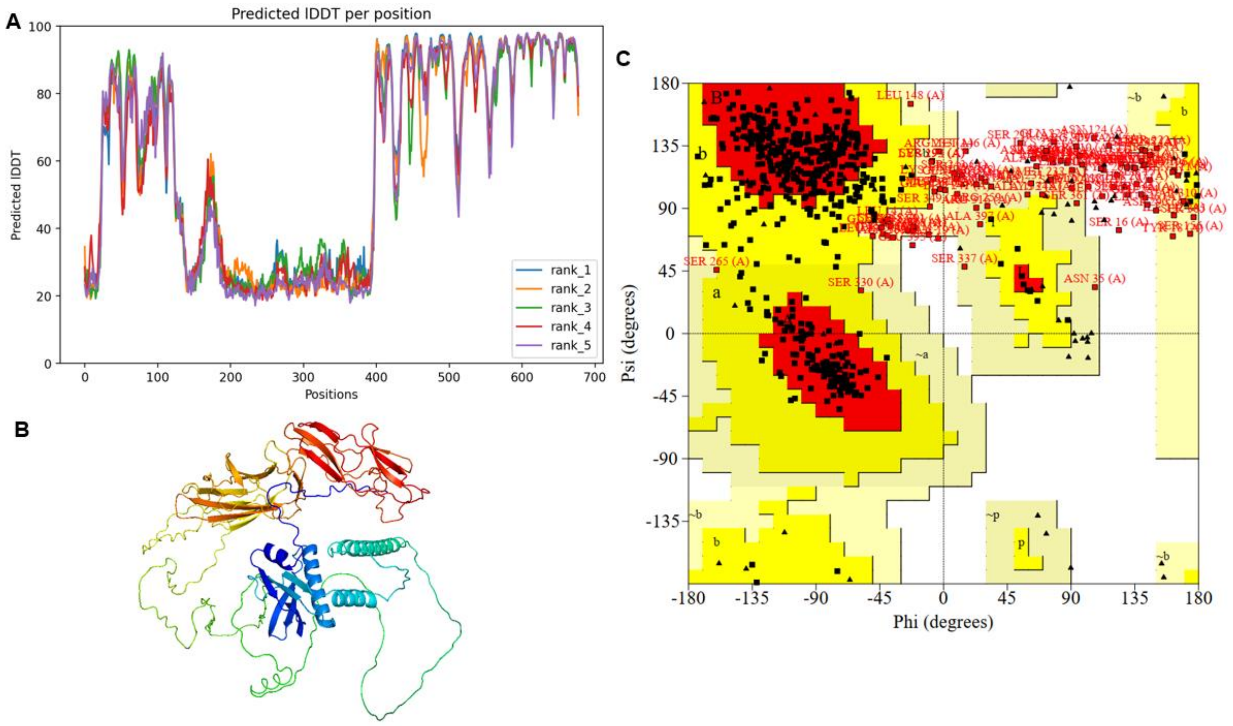

Figure S3. Prediction and validation of three-dimensional structure of the vaccine construct. (**A**) Predicted LDDT per residue for the 5 models obtained from AlphaFold Colab. (**B**) The 3D structure of rank_1 (model 2) representation in cartoon retrieved from PyMOL. (**C**) Ramachandran plot validation by PROCHECK tool.

Ranking models by 'plddt' metric:

- rank_1_model_2_ pLDDT=60.5
- rank_2_model_1_pLDDT=59.6
- rank_3_model_5_ pLDDT=59.5
- rank_4_model_3_ pLDDT=58.9
- rank_5_model_4_ pLDDT=58.8

II. Supplementary Table

Table S1. Sequence of the protein CARD8 (UniProt ID: Q9Y2G2).

```
>sp|Q9Y2G2|CARD8_HUMAN Caspase recruitment domain-containing protein 8
OS=Homo sapiens OX=9606 GN=CARD8 PE=1 SV=2
MEKKECPEKSSSSEEELPRRDSGSSRNIDASKLIRLQGSRKLLVDNSIRELQYTKTGIFF
QAEACVTNDTVYRELPCVSETLCDISHFFQEDDETEAEPLLFRAVPECQLSGGDIPSVSE
EQESSEGQDSGDICSEENQIVSSYASKVCFEIEEDYKNRQFLGPEGNVDVELIDKSTNRY
SVWFPTAGWYLWSATGLGFLVRDEVTVTIAFGSWSQHLALDLQHHEQWLVGGPLFDVTAE
PEEAVAEIHLPHFISLQAGEVDVSWFLVAHFKNEGMVLEHPARVEPFYAVLESPSFSLMG
ILLRIASGTRLSIPITSNTLIYYHPHPEDIKFHLYLVPSDALLTKAIDDEEDRFHGVRLQ
TSPPMEPLNFGSSYIVSNSANLKVMPKELKLSYRSPGEIQHFSKFYAGQMKEPIQLEITE
KRHGTLVWDTEVKPVDLQLVAASAPPPFSGAAFVKENHRQLQARMGDLKGVLDDLQDNEV
LTENEKELVEQEKTRQSKNEALLSMVEKKGDLALDVLFRSISERDPYLVSYLRQQNL
```

Table S2. Sequence of the protein NAIP (UniProt ID: Q13075).

```
>sp|Q13075|BIRC1_HUMAN Baculoviral IAP repeat-containing protein 1 OS=Homo
sapiens OX=9606 GN=NAIP PE=1 SV=3
MATQQKASDERISQFDHNLLPELSALLGLDAVQLAKELEEEEQKERAKMQKGYNSQMRSE
AKRLKTFVTYEPYSSWIPQEMAAAGFYFTGVKSGIQCFCCSLILFGAGLTRLPIEDHKRF
HPDCGFLLNKDVGNIAKYDIRVKNLKSRLRGGKMRYQEEEARLASFRNWPFYVQGISPCV
LSEAGFVFTGKQDTVQCFSCGGCLGNWEEGDDPWKEHAKWFPKCEFLRSKKSSEEITQYI
QSYKGFVDITGEHFVNSWVQRELPMASAYCNDSIFAYEELRLDSFKDWPRESAVGVAALA
KAGLFYTGIKDIVQCFSCGGCLEKWQEGDDPLDDHTRCFPNCPFLQNMKSSAEVTPDLQS
RGELCELLETTSESNLEDSIAVGPIVPEMAQGEAQWFQEAKNLNEQLRAAYTSASFRHMS
LLDISSDLATDHLLGCDLSIASKHISKPVQEPLVLPEVFGNLNSVMCVEGEAGSGKTVLL
KKIAFLWASGCCPLLNRFQLVFYLSLSSTRPDEGLASIICDQLLEKEGSVTEMCVRNIIQ
QLKNQVLFLLDDYKEICSIPQVIGKLIQKNHLSRTCLLIAVRTNRARDIRRYLETILEIK
AFPFYNTVCILRKLFSHNMTRLRKFMVYFGKNQSLQKIQKTPLFVAAICAHWFQYPFDPS
FDDVAVFKSYMERLSLRNKATAEILKATVSSCGELALKGFFSCCFEFNDDDLAEAGVDED
EDLTMCLMSKFTAQRLRPFYRFLSPAFQEFLAGMRLIELLDSDRQEHQDLGLYHLKQINS
PMMTVSAYNNFLNYVSSLPSTKAGPKIVSHLLHLVDNKESLENISENDDYLKHQPEISLQ
MQLLRGLWQICPQAYFSMVSEHLLVLALKTAYQSNTVAACSPFVLQFLQGRTLTLGALNL
QYFFDHPESLSLLRSIHFPIRGNKTSPRAHFSVLETCFDKSQVPTIDQDYASAFEPMNEW
ERNLAEKEDNVKSYMDMQRRASPDLSTGYWKLSPKQYKIPCLEVDVNDIDVVGQDMLEIL
MTVFSASQRIELHLNHSRGFIESIRPALELSKASVTKCSISKLELSAAEQELLLTLPSLE
SLEVSGTIQSQDQIFPNLDKFLCLKELSVDLEGNINVFSVIPEEFPNFHHMEKLLIQISA
EYDPSKLVKLIQNSPNLHVFHLKCNFFSDFGSLMTMLVSCKKLTEIKFSDSFFQAVPFVA
SLPNFISLKILNLEGQQFPDEETSEKFAYILGSLSNLEELILPTGDGIYRVAKLIIQQCQ
QLHCLRVLSFFKTLNDDSVVEIAKVAISGGFQKLENLKLSINHKITEEGYRNFFQALDNM
PNLQELDISRHFTECIKAQATTVKSLSQCVLRLPRLIRLNMLSWLLDADDIALLNVMKER
HPQSKYLTILQKWILPFSPIIQK
```

Table S3. Sequence of the protein NLRP1 (UniProt ID: Q9C000).

```
>sp|Q9C000|NLRP1_HUMAN NACHT, LRR and PYD domains-containing protein 1
OS=Homo sapiens OX=9606 GN=NLRP1 PE=1 SV=1
MAGGAWGRLACYLEFLKKEELKEFQLLLANKAHSRSSSGETPAQPEKTSGMEVASYLVAQ
YGEQRAWDLALHTWEQMGLRSLCAQAQEGAGHSPSFPYSPSEPHLGSPSQPTSTAVLMPW
IHELPAGCTQGSERRVLRQLPDTSGRRWREISASLLYQALPSSPDHESPSQESPNAPTST
AVLGSWGSPPQPSLAPREQEAPGTQWPLDETSGIYYTEIREREREKSEKGRPPWAAVVGT
PPQAHTSLQPHHHPWEPSVRESLCSTWPWKNEDFNQKFTQLLLLQRPHPRSQDPLVKRSW
PDYVEENRGHLIEIRDLFGPGLDTQEPRIVILQGAAGIGKSTLARQVKEAWGRGQLYGDR
FQHVFYFSCRELAQSKVVSLAELIGKDGTATPAPIRQILSRPERLLFILDGVDEPGWVLQ
EPSSELCLHWSQPQPADALLGSLLGKTILPEASFLITARTTALQNLIPSLEQARWVEVLG
FSESSRKEYFYRYFTDERQAIRAFRLVKSNKELWALCLVPWVSWLACTCLMQQMKRKEKL
TLTSKTTTTLCLHYLAQALQAQPLGPQLRDLCSLAAEGIWQKKTLFSPDDLRKHGLDGAI
ISTFLKMGILQEHPIPLSYSFIHLCFQEFFAAMSYVLEDEKGRGKHSNCIIDLEKTLEAY
GIHGLFGASTTRFLLGLLSDEGEREMENIFHCRLSQGRNLMQWVPSLQLLLQPHSLESLH
CLYETRNKTFLTQVMAHFEEMGMCVETDMELLVCTFCIKFSRHVKKLQLIEGRQHRSTWS
PTMVVLFRWVPVTDAYWQILFSVLKVTRNLKELDLSGNSLSHSAVKSLCKTLRRPRCLLE
TLRLAGCGLTAEDCKDLAFGLRANQTLTELDLSFNVLTDAGAKHLCQRLRQPSCKLQRLQ
LVSCGLTSDCCQDLASVLSASPSLKELDLQQNNLDDVGVRLLCEGLRHPACKLIRLGLDQ
TTLSDEMRQELRALEQEKPQLLIFSRRKPSVMTPTEGLDTGEMSNSTSSLKRQRLGSERA
ASHVAQANLKLLDVSKIFPIAEIAEESSPEVVPVELLCVPSPASQGDLHTKPLGTDDDFW
GPTGPVATEVVDKEKNLYRVHFPVAGSYRWPNTGLCFVMREAVTVEIEFCVWDQFLGEIN
PQHSWMVAGPLLDIKAEPGAVEAVHLPHFVALQGGHVDTSLFQMAHFKEEGMLLEKPARV
ELHHIVLENPSFSPLGVLLKMIHNALRFIPVTSVVLLYHRVHPEEVTFHLYLIPSDCSIR
KAIDDLEMKFQFVRIHKPPPLTPLYMGCRYTVSGSGSGMLEILPKELELCYRSPGEDQLF
SEFYVGHLGSGIRLQVKDKKDETLVWEALVKPGDLMPATTLIPPARIAVPSPLDAPQLLH
FVDQYREQLIARVTSVEVVLDKLHGQVLSQEQYERVLAENTRPSQMRKLFSLSQSWDRKC
KDGLYQALKETHPHLIMELWEKGSKKGLLPLSS
```

Table S4. Sequence of the protein NLRP3 (UniProt ID: Q96P20).

```
>sp|Q96P20|NLRP3_HUMAN NACHT, LRR and PYD domains-containing protein 3
OS=Homo sapiens OX=9606 GN=NLRP3 PE=1 SV=3
MKMASTRCKLARYLEDLEDVDLKKFKMHLEDYPPQKGCIPLPRGQTEKADHVDLATLMID
FNGEEKAWAMAVWIFAAINRRDLYEKAKRDEPKWGSDNARVSNPTVICQEDSIEEEWMGL
LEYLSRISICKMKKDYRKKYRKYVRSRFQCIEDRNARLGESVSLNKRYTRLRLIKEHRSQ
QEREQELLAIGKTKTCESPVSPIKMELLFDPDDEHSEPVHTVVFQGAAGIGKTILARKMM
LDWASGTLYQDRFDYLFYIHCREVSLVTQRSLGDLIMSCCPDPNPPIHKIVRKPSRILFL
MDGFDELQGAFDEHIGPLCTDWQKAERGDILLSSLIRKKLLPEASLLITTRPVALEKLQH
LLDHPRHVEILGFSEAKRKEYFFKYFSDEAQARAAFSLIQENEVLFTMCFIPLVCWIVCT
GLKQQMESGKSLAQTSKTTTAVYVFFLSSLLQPRGGSQEHGLCAHLWGLCSLAADGIWNQ
KILFEESDLRNHGLQKADVSAFLRMNLFQKEVDCEKFYSFIHMTFQEFFAAMYYLLEEEK
EGRTNVPGSRLKLPSRDVTVLLENYGKFEKGYLIFVVRFLFGLVNQERTSYLEKKLSCKI
SQQIRLELLKWIEVKAKAKKLQIQPSQLELFYCLYEMQEEDFVQRAMDYFPKIEINLSTR
MDHMVSSFCIENCHRVESLSLGFLHNMPKEEEEEEKEGRHLDMVQCVLPSSSHAACSHGL
VNSHLTSSFCRGLFSVLSTSQSLTELDLSDNSLGDPGMRVLCETLQHPGCNIRRLWLGRC
GLSHECCFDISLVLSSNQKLVELDLSDNALGDFGIRLLCVGLKHLLCNLKKLWLVSCCLT
SACCQDLASVLSTSHSLTRLYVGENALGDSGVAILCEKAKNPQCNLQKLGLVNSGLTSVC
CSALSSVLSTNQNLTHLYLRGNTLGDKGIKLLCEGLLHPDCKLQVLELDNCNLTSHCCWD
LSTLLTSSQSLRKLSLGNNDLGDLGVMMFCEVLKQQSCLLQNLGLSEMYFNYETKSALET
LQEEKPELTVVFEPSW
```

Table S5. Structure information of 5 models predicted by I-TASSER.

**Model**	**C-score**	**Exp. TM-Score**	**Exp. RMSD**	**No. of decoys**	**Cluster density**
Model 1	-2.10	0.46±0.15	13.2±4.1Å	128	0.0410
Model 2	-2.10	-	-	126	0.0412
Model 3	-2.35	-	-	114	0.0322
Model 4	-4.11	-	-	112	0.0055
Model 5	-4.12	-	-	102	0.0055

Table S6. Structure information of 5 refined models obtained from GalaxyRefine.

**Model**	**GDT-HA**	**RMSD**	**MolProbity**	**Clash score**	**Poor rotamers**	**Rama favored**
Initial*	1.0000	0.000	3.097	7.6	13.1	71.3
MODEL 1	0.9218	0.483	2.321	16.8	0.4	88.2
MODEL 2	0.9314	0.459	2.260	16.1	0.7	89.9
MODEL 3	0.9399	0.448	2.390	17.1	1.3	88.9
MODEL 4	0.9314	0.464	2.309	15.8	1.1	88.9
MODEL 5	0.9310	0.468	2.276	15.6	0.5	88.8

*Initial is the 3D structure which was predicted by I-TASSER (Model 1 shown in Table S5)

Table S7. Predicted disulfide bonds with corresponding residue pairs, angles, and energy values, using Disulfide by Design 2 v2.13 server.

No.	Res.1 Seq. #	Res. 1 aa	Res.2 Seq. #	Res. 2 aa	χ3 Angle (°)	Energy (kcal/mol)
1	6	PHE	567	VAL	-107.81	5.72
2	9	PHE	672	GLY	111.76	4.05
3	23	PRO	28	ASP	78.34	1.37
4	23	PRO	29	LEU	-97.31	1
5	33	TYR	36	THR	116.32	3.63
6	45	ILE	97	TYR	121.74	4.69
7	57	GLU	73	VAL	-90.22	8.24
8	74	PRO	78	HIS	99.54	1.34
9	96	ALA	101	ALA	-106.67	4.27
10	101	ALA	127	ALA	-88.83	2.25
11	104	GLU	123	ALA	93.58	5
12	105	LYS	121	SER	99.66	4.28
13	107	CYS	118	ALA	-104.86	3.82
14	107	CYS	119	ALA	90.7	4.88
15	112	LYS	116	ALA	123.14	4.18
16	416	SER	544	VAL	-106.53	3.91
17	422	ASN	550	ASN	113.69	4.07
18	431	GLN	507	TYR	-97.99	5.32
19	438	SER	501	PRO	-116.41	5.17
20	453	ASP	494	TYR	117.73	8.04
21	454	TYR	491	ARG	-110.42	6.09
22	455	VAL	492	VAL	-106.04	7.55
23	458	GLN	529	ILE	-101.43	3.39
24	458	GLN	531	ARG	120.66	5.06
25	461	SER	485	THR	111.45	3.14
26	463	TYR	467	LEU	89.7	3.41
27	464	GLY	524	LEU	-95.31	0.47
28	472	GLY	483	PHE	-105.48	6.37
29	512	SER	558	GLY	-110.49	8.29
30	513	SER	675	PHE	125.32	5.24
31	514	ALA	560	CYS	-93.56	2.53
32	514	ALA	675	PHE	85.6	5.44
33	516	GLY	554	VAL	109.17	3.87
34	530	LEU	543	PHE	-80.1	7.43
35	583	THR	646	ALA	88.11	4.96
36	601	ALA	658	THR	114.3	3.46
37	602	ASP	607	ILE	107.47	2.13
38	607	ILE	621	GLY	-116.42	5.23
39	608	PHE	669	SER	122.94	3.96
40	612	ALA	666	ASN	-97.19	0.68
41	612	ALA	668	GLN	109.07	2.07
42	622	VAL	669	SER	101.1	1.35
43	623	GLN	656	ALA	80.16	2.97

Table S8. Docking result of vaccine with TLR2, generated by ClusPro v2.0 server.

Cluster	Members	Representative	Weighted Score
**0**	73	Center	-1059.9
		Lowest Energy	-1193.2
**1**	53	Center	-1218.7
		Lowest Energy	-1252.7
**2**	50	Center	-1096.8
		Lowest Energy	-1417.7
**3**	40	Center	-1309.7
		Lowest Energy	-1475.0
**4**	37	Center	-1043.9
		Lowest Energy	-1169.7
**5**	33	Center	-1101.1
		Lowest Energy	-1364.3
**6**	32	Center	-1257.3
		Lowest Energy	-1258.2
**7**	31	Center	-1269.3
		Lowest Energy	-1364.7
**8**	31	Center	-1276.0
		Lowest Energy	-1276.0
**9**	25	Center	-1023.5
		Lowest Energy	-1130.7
**10**	23	Center	-1016.6
		Lowest Energy	-1124.9
**11**	21	Center	-1287.2
		Lowest Energy	-1287.2
**12**	19	Center	-1249.3
		Lowest Energy	-1249.3
**13**	18	Center	-1082.8
		Lowest Energy	-1094.4
**14**	17	Center	-1024.3
		Lowest Energy	-1088.1

Cluster	Members	Representative	Weighted Score
**15**	16	Center	-1097.1
		Lowest Energy	-1201.7
**16**	16	Center	-1182.4
		Lowest Energy	-1182.4
**17**	15	Center	-1093.6
		Lowest Energy	-1134.1
**18**	15	Center	-995.2
		Lowest Energy	-1193.4
**19**	14	Center	-1044.9
		Lowest Energy	-1264.6
**20**	14	Center	-1157.1
		Lowest Energy	-1366.7
**21**	14	Center	-1053.2
		Lowest Energy	-1483.1
**22**	13	Center	-1022.3
		Lowest Energy	-1063.7
**23**	12	Center	-1380.8
		Lowest Energy	-1380.8
**24**	10	Center	-1128.6
		Lowest Energy	-1210.5
**25**	10	Center	-1032.9
		Lowest Energy	-1095.7
**26**	10	Center	-1094.6
		Lowest Energy	-1097.5
**27**	10	Center	-1015.9
		Lowest Energy	-1122.7
**28**	7	Center	-1000.7
		Lowest Energy	-1017.2
**29**	7	Center	-1105.0
		Lowest Energy	-1337.9

Table S9. Docking result of vaccine with TLR4, generated by ClusPro v2.0 server.

Cluster	Members	Representative	Weighted Score
**0**	44	Center	-1362.4
		Lowest Energy	-1468.3
**1**	41	Center	-1090.4
		Lowest Energy	-1301.8
**2**	37	Center	-1039.9
		Lowest Energy	-1284.8
**3**	31	Center	-1083.1
		Lowest Energy	-1222.4
**4**	28	Center	-1077.6
		Lowest Energy	-1206.0
**5**	27	Center	-1134.5
		Lowest Energy	-1187.7
**6**	26	Center	-1118.2
		Lowest Energy	-1194.7
**7**	25	Center	-1100.6
		Lowest Energy	-1266.2
**8**	23	Center	-1149.5
		Lowest Energy	-1190.8
**9**	20	Center	-1063.9
		Lowest Energy	-1195.3
**10**	19	Center	-1080.1
		Lowest Energy	-1113.7
**11**	19	Center	-1004.3
		Lowest Energy	-1150.4
**12**	18	Center	-994.8
		Lowest Energy	-1111.7
**13**	18	Center	-1137.0
		Lowest Energy	-1147.8
**14**	18	Center	-1117.7
		Lowest Energy	-1213.3

Cluster	Members	Representative	Weighted Score
**15**	17	Center	-1170.4
		Lowest Energy	-1247.6
**16**	15	Center	-988.2
		Lowest Energy	-1265.0
**17**	14	Center	-1127.2
		Lowest Energy	-1127.2
**18**	14	Center	-1078.8
		Lowest Energy	-1314.8
**19**	13	Center	-1018.2
		Lowest Energy	-1101.8
**20**	13	Center	-1140.3
		Lowest Energy	-1140.3
**21**	13	Center	-1077.2
		Lowest Energy	-1129.7
**22**	12	Center	-1119.3
		Lowest Energy	-1119.3
**23**	12	Center	-1106.6
		Lowest Energy	-1106.6
**24**	12	Center	-1062.1
		Lowest Energy	-1069.5
**25**	11	Center	-1044.7
		Lowest Energy	-1083.8
**26**	10	Center	-1008.7
		Lowest Energy	-1214.6
**27**	10	Center	-1043.1
		Lowest Energy	-1043.1
**28**	10	Center	-1139.2
		Lowest Energy	-1245.7
**29**	10	Center	-1169.3
		Lowest Energy	-1169.3

Table S10. Docking result of vaccine with TLR5, generated by ClusPro v2.0 server.

Cluster	Members	Representative	Weighted Score
**0**	66	Center	-1302.7
		Lowest Energy	-1916.8
**1**	36	Center	-1208.3
		Lowest Energy	-1263.0
**2**	32	Center	-1290.8
		Lowest Energy	-1554.4
**3**	32	Center	-1226.2
		Lowest Energy	-1400.6
**4**	25	Center	-1177.3
		Lowest Energy	-1361.9
**5**	24	Center	-1340.6
		Lowest Energy	-1465.6
**6**	24	Center	-1430.8
		Lowest Energy	-1430.8
**7**	23	Center	-1172.9
		Lowest Energy	-1357.9
**8**	21	Center	-1134.2
		Lowest Energy	-1434.5
**9**	20	Center	-1200.3
		Lowest Energy	-1452.4
**10**	17	Center	-1290.4
		Lowest Energy	-1441.4
**11**	17	Center	-1216.2
		Lowest Energy	-1231.2
**12**	17	Center	-1140.4
		Lowest Energy	-1423.5
**13**	17	Center	-1244.3
		Lowest Energy	-1419.6
**14**	17	Center	-1159.6
		Lowest Energy	-1473.7

Cluster	Members	Representative	Weighted Score
**15**	16	Center	-1392.6
		Lowest Energy	-1392.6
**16**	15	Center	-1301.8
		Lowest Energy	-1499.5
**17**	15	Center	-1236.1
		Lowest Energy	-1532.6
**18**	14	Center	-1184.9
		Lowest Energy	-1267.7
**19**	14	Center	-1171.0
		Lowest Energy	-1285.8
**20**	14	Center	-1159.6
		Lowest Energy	-1295.2
**21**	14	Center	-1520.3
		Lowest Energy	-1520.3
**22**	14	Center	-1195.9
		Lowest Energy	-1617.0
**23**	13	Center	-1258.3
		Lowest Energy	-1386.9
**24**	12	Center	-1215.6
		Lowest Energy	-1215.6
**25**	12	Center	-1331.1
		Lowest Energy	-1331.1
**26**	12	Center	-1134.6
		Lowest Energy	-1268.8
**27**	11	Center	-1169.8
		Lowest Energy	-1320.3
**28**	11	Center	-1341.6
		Lowest Energy	-1404.1
**29**	11	Center	-1209.1
		Lowest Energy	-1457.8

Table S11. Docking result of vaccine with TLR3, generated by ClusPro v2.0 server.

Cluster	Members	Representative	Weighted Score
**0**	51	Center	-1076.1
		Lowest Energy	-1364.1
**1**	37	Center	-1054.7
		Lowest Energy	-1112.7
**2**	35	Center	-1075.4
		Lowest Energy	-1111.3
**3**	23	Center	-972.3
		Lowest Energy	-1171.7
**4**	23	Center	-1062.4
		Lowest Energy	-1257.4
**5**	23	Center	-1026.3
		Lowest Energy	-1175.1
**6**	22	Center	-1010.3
		Lowest Energy	-1125.7
**7**	20	Center	-1002.1
		Lowest Energy	-1209.6
**8**	19	Center	-1007.1
		Lowest Energy	-1080.5
**9**	19	Center	-1039.1
		Lowest Energy	-1194.3
**10**	17	Center	-1063.8
		Lowest Energy	-1455.3
**11**	17	Center	-971.0
		Lowest Energy	-1067.9
**12**	16	Center	-1192.7
		Lowest Energy	-1272.6
**13**	16	Center	-1088.6
		Lowest Energy	-1088.6
**14**	15	Center	-980.2
		Lowest Energy	-1111.3

Cluster	Members	Representative	Weighted Score
**15**	14	Center	-1012.8
		Lowest Energy	-1263.1
**16**	14	Center	-1097.3
		Lowest Energy	-1097.3
**17**	13	Center	-1014.3
		Lowest Energy	-1099.9
**18**	13	Center	-980.6
		Lowest Energy	-1196.2
**19**	12	Center	-1011.0
		Lowest Energy	-1127.7
**20**	12	Center	-1048.1
		Lowest Energy	-1254.9
**21**	12	Center	-1026.6
		Lowest Energy	-1182.8
**22**	12	Center	-1048.2
		Lowest Energy	-1095.1
**23**	11	Center	-982.3
		Lowest Energy	-1047.1
**24**	11	Center	-1336.6
		Lowest Energy	-1336.6
**25**	11	Center	-1247.9
		Lowest Energy	-1247.9
**26**	10	Center	-966.9
		Lowest Energy	-1087.9
**27**	10	Center	-1020.0
		Lowest Energy	-1101.6
**28**	10	Center	-1126.9
		Lowest Energy	-1172.0
**29**	10	Center	-1095.2
		Lowest Energy	-1254.1

Table S12. Docking result of vaccine with TLR7, generated by ClusPro v2.0 server.

Cluster	Members	Representative	Weighted Score
**0**	40	Center	-1128.3
		Lowest Energy	-1335.4
**1**	32	Center	-1343.8
		Lowest Energy	-1343.8
**2**	31	Center	-1506.7
		Lowest Energy	-1663.4
**3**	24	Center	-1215.4
		Lowest Energy	-1356.0
**4**	24	Center	-1579.2
		Lowest Energy	-1579.2
**5**	22	Center	-1210.7
		Lowest Energy	-1210.7
**6**	22	Center	-1184.9
		Lowest Energy	-1184.9
**7**	22	Center	-1138.8
		Lowest Energy	-1327.6
**8**	21	Center	-1285.0
		Lowest Energy	-1387.2
**9**	21	Center	-1264.0
		Lowest Energy	-1292.0
**10**	21	Center	-1211.6
		Lowest Energy	-1249.3
**11**	20	Center	-1068.5
		Lowest Energy	-1197.6
**12**	19	Center	-1198.1
		Lowest Energy	-1294.1
**13**	17	Center	-1241.9
		Lowest Energy	-1241.9
**14**	16	Center	-1206.1
		Lowest Energy	-1206.1

Cluster	Members	Representative	Weighted Score
**15**	15	Center	-1211.1
		Lowest Energy	-1211.1
**16**	14	Center	-1182.5
		Lowest Energy	-1217.4
**17**	14	Center	-1077.3
		Lowest Energy	-1151.1
**18**	13	Center	-1204.0
		Lowest Energy	-1204.0
**19**	13	Center	-1116.7
		Lowest Energy	-1251.7
**20**	13	Center	-1101.5
		Lowest Energy	-1147.7
**21**	12	Center	-1240.9
		Lowest Energy	-1324.3
**22**	12	Center	-1219.5
		Lowest Energy	-1353.3
**23**	12	Center	-1186.6
		Lowest Energy	-1186.6
**24**	12	Center	-1127.8
		Lowest Energy	-1191.3
**25**	12	Center	-1118.0
		Lowest Energy	-1166.0
**26**	12	Center	-1069.1
		Lowest Energy	-1181.8
**27**	11	Center	-1115.9
		Lowest Energy	-1127.4
**28**	11	Center	-1151.1
		Lowest Energy	-1220.1
**29**	10	Center	-1371.6
		Lowest Energy	-1371.6

Table S13. Docking result of vaccine with TLR8, generated by ClusPro v2.0 server.

Cluster	Members	Representative	Weighted Score
**0**	54	Center	-1393.8
		Lowest Energy	-1431.7
**1**	46	Center	-1289.0
		Lowest Energy	-1512.4
**2**	44	Center	-1242.1
		Lowest Energy	-1403.1
**3**	37	Center	-1248.9
		Lowest Energy	-1343.8
**4**	35	Center	-1169.2
		Lowest Energy	-1435.5
**5**	28	Center	-1203.7
		Lowest Energy	-1373.9
**6**	27	Center	-1275.2
		Lowest Energy	-1288.5
**7**	25	Center	-1118.1
		Lowest Energy	-1647.3
**8**	22	Center	-1503.8
		Lowest Energy	-1635.0
**9**	22	Center	-1244.3
		Lowest Energy	-1378.2
**10**	21	Center	-1288.5
		Lowest Energy	-1477.5
**11**	19	Center	-1222.1
		Lowest Energy	-1434.5
**12**	19	Center	-1138.3
		Lowest Energy	-1373.8
**13**	18	Center	-1501.1
		Lowest Energy	-1501.1
**14**	18	Center	-1145.9
		Lowest Energy	-1274.5

Cluster	Members	Representative	Weighted Score
**15**	17	Center	-1321.0
		Lowest Energy	-1440.4
**16**	17	Center	-1182.8
		Lowest Energy	-1438.4
**17**	16	Center	-1378.7
		Lowest Energy	-1442.6
**18**	15	Center	-1183.3
		Lowest Energy	-1316.8
**19**	15	Center	-1606.2
		Lowest Energy	-1606.2
**20**	15	Center	-1165.2
		Lowest Energy	-1435.2
**21**	14	Center	-1254.6
		Lowest Energy	-1277.2
**22**	13	Center	-1231.1
		Lowest Energy	-1390.7
**23**	12	Center	-1143.8
		Lowest Energy	-1343.1
**24**	12	Center	-1449.4
		Lowest Energy	-1534.3
**25**	12	Center	-1149.4
		Lowest Energy	-1449.2
**26**	12	Center	-1131.4
		Lowest Energy	-1304.7
**27**	12	Center	-1136.5
		Lowest Energy	-1199.3
**28**	12	Center	-1114.4
		Lowest Energy	-1288.2
**29**	11	Center	-1152.9
		Lowest Energy	-1231.3

Table S14. List of atom-atom interactions across vaccine-TLR2 interface.

Hydrogen bonds

	<----- **TLR2 ATOM** ----->						<----- **Vaccine ATOM** ----->					
	**Atom no.**	**Atom name**	**Res name**	**Res no.**	**Chain**		**Atom no.**	**Atom name**	**Res name**	**Res no.**	**Chain**	**Distance**
1.	1042	NZ	LYS	137	A	<-->	7725	O	GLY	246	B	2.45
2.	1042	NZ	LYS	137	A	<-->	7732	OG	SER	247	B	2.66
3.	1739	NZ	LYS	208	A	<-->	8214	OE1	GLU	297	B	2.70
4.	2387	O	ASN	274	A	<-->	8876	N	GLY	371	B	2.69
5.	2592	NH1	ARG	296	A	<-->	9072	O	LEU	392	B	2.64
6.	2617	O	ASP	299	A	<-->	9085	NE2	GLN	393	B	2.78
7.	2620	OD1	ASP	299	A	<-->	9090	N	ILE	394	B	3.00
8.	2630	OD1	ASN	300	A	<-->	9109	N	ALA	396	B	3.13
9.	2630	OD1	ASN	300	A	<-->	9558	OH	TYR	447	B	2.79
10.	2629	ND2	ASN	300	A	<-->	9112	O	ALA	396	B	2.98
11.	2629	ND2	ASN	300	A	<-->	9124	O	ALA	398	B	2.88
12.	2653	NH2	ARG	302	A	<-->	9575	OE1	GLU	449	B	2.68
13.	2683	OD1	ASP	305	A	<-->	8881	N	GLY	372	B	3.07
14.	2684	OD2	ASP	305	A	<-->	8894	N	VAL	374	B	2.93
15.	2832	O	PRO	320	A	<-->	8450	N	GLY	324	B	2.97
16.	2856	O	PHE	322	A	<-->	8772	N	SER	361	B	3.04
17.	2891	O	PHE	325	A	<-->	8780	N	GLU	362	B	3.15
18.	3017	NH1	ARG	337	A	<-->	8884	O	GLY	372	B	2.72
19.	3018	NH2	ARG	337	A	<-->	8884	O	GLY	372	B	2.83
20.	3018	NH2	ARG	337	A	<-->	8889	O	SER	373	B	2.76
21.	3106	OD1	ASN	345	A	<-->	8406	NE	ARG	319	B	2.74
22.	3106	OD1	ASN	345	A	<-->	8409	NH2	ARG	319	B	2.64
23.	3113	O	SER	346	A	<-->	8797	NE2	GLN	363	B	2.67
24.	3126	NZ	LYS	347	A	<-->	8402	O	ARG	319	B	2.67
25.	3126	NZ	LYS	347	A	<-->	8419	O	LEU	320	B	2.45
26.	3126	NZ	LYS	347	A	<-->	8432	OD1	ASN	321	B	2.65
27.	3154	O	LEU	350	A	<-->	8818	NH2	ARG	365	B	2.75
28.	3249	NZ	LYS	360	A	<-->	8940	O	LYS	378	B	2.49
29.	3249	NZ	LYS	360	A	<-->	8967	OE2	GLU	380	B	2.74
30.	3337	OE1	GLU	369	A	<-->	8409	NH2	ARG	319	B	2.77
31.	3472	OE1	GLU	383	A	<-->	8997	N	SER	385	B	2.96
32.	3472	OE1	GLU	383	A	<-->	9002	OG	SER	385	B	2.86
33.	3482	OD2	ASP	384	A	<-->	9013	NZ	LYS	386	B	2.90
34.	3610	O	ASN	397	A	<-->	8371	NE	ARG	316	B	2.77
35.	3610	O	ASN	397	A	<-->	8374	NH2	ARG	316	B	2.70
36.	3633	O	LEU	399	A	<-->	8374	NH2	ARG	316	B	2.69
37.	3862	OD1	ASN	423	A	<-->	8374	NH2	ARG	316	B	2.80
38.	4103	NE	ARG	447	A	<-->	8345	OG	SER	313	B	2.88
39.	4106	NH2	ARG	447	A	<-->	8351	O	LEU	314	B	2.66
40.	4277	ND2	ASN	466	A	<-->	8338	O	GLY	312	B	2.94
41.	4482	NH1	ARG	486	A	<-->	8333	O	GLY	311	B	2.63
42.	4483	NH2	ARG	486	A	<-->	8308	O	ARG	309	B	2.63

Salt bridges

	TLR2 ATOM						Vaccine ATOM					
	Atom no.	Atom name	Res name	Res no.	Chain		Atom no.	Atom name	Res name	Res no.	Chain	Distance
1.	1739	NZ	LYS	208	A	<-->	8215	OE2	GLU	297	B	2.70
2.	2653	NH2	ARG	302	A	<-->	9575	OE1	GLU	449	B	2.68
3.	3005	OE1	GLU	336	A	<-->	8932	NZ	LYS	377	B	2.65
4.	3249	NZ	LYS	360	A	<-->	8966	OE1	GLU	380	B	2.74
5.	3338	OE2	GLU	369	A	<-->	8409	NH2	ARG	319	B	2.77
6.	3482	OD2	ASP	384	A	<-->	9013	NZ	LYS	386	B	2.90

Table S15. List of atom-atom interactions across vaccine-TLR4 interface.

Hydrogen bonds

	<----- TLR4 ATOM ----->						<----- Vaccine ATOM ----->					
	**Atom no.**	**Atom name**	**Res name**	**Res no.**	**Chain**		**Atom no.**	**Atom name**	**Res name**	**Res no.**	**Chain**	**Distance**
1.	2501	NH1	ARG	289	A	<-->	8723	O	VAL	310	B	2.69
2.	2501	NH1	ARG	289	A	<-->	8731	O	GLY	311	B	2.62
3.	4278	O	SER	471	A	<-->	8293	NH2	ARG	261	B	2.68
4.	4406	O	GLU	485	A	<-->	8951	N	SER	337	B	3.20
5.	4410	OE1	GLU	485	A	<-->	8956	OG	SER	337	B	2.80
6.	4420	OD1	ASN	486	A	<-->	8968	NH1	ARG	338	B	2.66
7.	4519	NH1	ARG	496	A	<-->	8286	O	ARG	261	B	2.65
8.	4519	NH1	ARG	496	A	<-->	8303	O	GLY	262	B	2.62
9.	4530	O	ASN	497	A	<-->	8267	NH2	ARG	259	B	2.82
10.	4534	OD1	ASN	497	A	<-->	8290	NE	ARG	261	B	3.03
11.	4534	OD1	ASN	497	A	<-->	8293	NH2	ARG	261	B	2.66
12.	4610	NE2	GLN	505	A	<-->	8826	O	ASN	321	B	2.81
13.	4630	OE1	GLN	507	A	<-->	8969	NH2	ARG	338	B	2.58
14.	4651	OE2	GLU	509	A	<-->	8941	N	GLY	335	B	2.86
15.	4651	OE2	GLU	509	A	<-->	8946	N	GLY	336	B	3.01
16.	4719	O	ASN	517	A	<-->	8310	N	GLY	264	B	3.06
17.	4749	OG	SER	520	A	<-->	8277	O	LEU	260	B	2.67
18.	4922	O	PHE	538	A	<-->	8348	OH	TYR	268	B	2.71
19.	4960	NZ	LYS	541	A	<-->	8331	O	LEU	267	B	2.63
20.	5072	O	LEU	553	A	<-->	8858	OG	SER	325	B	2.73
21.	5169	NE2	GLN	562	A	<-->	8471	O	GLY	281	B	2.87
22.	5169	NE2	GLN	562	A	<-->	8476	O	GLY	282	B	3.00
23.	5420	OE1	GLN	588	A	<-->	8501	N	LEU	286	B	2.79
24.	5460	NE2	GLN	592	A	<-->	8545	OD2	ASP	290	B	3.12
25.	5498	NZ	LYS	595	A	<-->	8541	O	ASP	290	B	2.49
26.	5498	NZ	LYS	595	A	<-->	8544	OD1	ASP	290	B	2.78
27.	5509	OD1	ASP	596	A	<-->	8562	NZ	LYS	292	B	2.53
28.	5527	O	ARG	598	A	<-->	8583	NE2	GLN	294	B	2.84
29.	5531	NE	ARG	598	A	<-->	8584	OE1	GLN	294	B	2.85
30.	5533	NH1	ARG	598	A	<-->	8557	O	LYS	292	B	2.66
31.	5533	NH1	ARG	598	A	<-->	8570	O	LEU	293	B	2.59
32.	5534	NH2	ARG	598	A	<-->	8584	OE1	GLN	294	B	2.67
33.	5579	N	GLU	603	A	<-->	8599	O	LEU	296	B	2.86
34.	5604	OE1	GLU	605	A	<-->	8596	N	LEU	296	B	3.01
35.	5746	O	LEU	621	A	<-->	8554	N	LYS	292	B	3.18

Salt bridges

	<----- TLR4 ATOM ----->						<----- Vaccine ATOM ----->					
	**Atom no.**	**Atom name**	**Res name**	**Res no.**	**Chain**		**Atom no.**	**Atom name**	**Res name**	**Res no.**	**Chain**	**Distance**
1.	4308	OE2	GLU	474	A	<-->	8267	NH2	ARG	259	B	2.71
2.	5498	NZ	LYS	595	A	<-->	8545	OD2	ASP	290	B	2.78
3.	5509	OD1	ASP	596	A	<-->	8562	NZ	LYS	292	B	2.53

Table S16. List of atom-atom interactions across vaccine-TLR5 interface.

Hydrogen bonds

	<----- TLR5 ATOM ----->						<----- Vaccine ATOM ----->					
	**Atom no.**	**Atom name**	**Res name**	**Res no.**	**Chain**		**Atom no.**	**Atom name**	**Res name**	**Res no.**	**Chain**	**Distance**
1.	40	N	PHE	26	A	<-->	6492	OG	SER	51	B	3.01
2.	75	NH1	ARG	29	A	<-->	8623	O	THR	274	B	2.66
3.	121	OH	TYR	33	A	<-->	6495	N	LEU	52	B	2.95
4.	133	NH1	ARG	34	A	<-->	8681	O	MET	280	B	2.63
5.	133	NH1	ARG	34	A	<-->	8690	O	GLY	281	B	2.66
6.	156	O	CYS	36	A	<-->	6538	NH2	ARG	56	B	2.83
7.	167	OD1	ASN	37	A	<-->	8720	N	LEU	286	B	2.82
8.	186	OG1	THR	39	A	<-->	8763	OD1	ASP	290	B	2.81
9.	197	OE1	GLN	40	A	<-->	8781	NZ	LYS	292	B	2.62
10.	281	OE1	GLU	49	A	<-->	8626	OG1	THR	274	B	2.89
11.	291	NE	ARG	50	A	<-->	8591	O	LEU	271	B	3.00
12.	293	NH1	ARG	50	A	<-->	8632	O	HIS	275	B	2.65
13.	293	NH1	ARG	50	A	<-->	8644	O	PRO	276	B	3.11
14.	293	NH1	ARG	50	A	<-->	8651	O	HIS	277	B	2.64
15.	294	NH2	ARG	50	A	<-->	8591	O	LEU	271	B	2.69
16.	294	NH2	ARG	50	A	<-->	8600	O	LYS	272	B	2.68
17.	294	NH2	ARG	50	A	<-->	8626	OG1	THR	274	B	2.77
18.	294	NH2	ARG	50	A	<-->	8632	O	HIS	275	B	2.67
19.	370	OH	TYR	57	A	<-->	8723	O	LEU	286	B	2.77
20.	391	NH1	ARG	59	A	<-->	8760	O	ASP	290	B	2.67
21.	391	NH1	ARG	59	A	<-->	8771	SG	CYS	291	B	3.00
22.	392	NH2	ARG	59	A	<-->	8760	O	ASP	290	B	2.76
23.	434	O	SER	64	A	<-->	8360	NZ	LYS	248	B	2.70
24.	436	OG	SER	64	A	<-->	8407	NH2	ARG	252	B	2.70
25.	656	OD2	ASP	87	A	<-->	8802	NE2	GLN	294	B	2.93
26.	706	NE	ARG	92	A	<-->	8417	O	VAL	253	B	2.78
27.	709	NH2	ARG	92	A	<-->	8400	O	ARG	252	B	2.79
28.	709	NH2	ARG	92	A	<-->	8417	O	VAL	253	B	3.31
29.	722	ND2	ASN	93	A	<-->	8386	OD2	ASP	250	B	3.21
30.	772	NH1	ARG	98	A	<-->	8550	O	LEU	267	B	3.35
31.	845	NZ	LYS	106	A	<-->	9097	O	LEU	327	B	2.60
32.	845	NZ	LYS	106	A	<-->	9106	O	ILE	328	B	2.56
33.	1019	OE2	GLU	123	A	<-->	8567	OH	TYR	268	B	2.77
34.	1040	NH2	ARG	125	A	<-->	8567	OH	TYR	268	B	3.14
35.	1108	OG	SER	132	A	<-->	9077	OG	SER	325	B	3.31
36.	1241	NZ	LYS	145	A	<-->	8449	O	LEU	256	B	2.65
37.	1241	NZ	LYS	145	A	<-->	8458	O	LYS	257	B	2.63
38.	1241	NZ	LYS	145	A	<-->	8471	O	SER	258	B	2.51
39.	1267	OG1	THR	148	A	<-->	8529	N	GLY	264	B	3.15
40.	1354	OE1	GLN	156	A	<-->	9080	N	TYR	326	B	2.84
41.	1353	NE2	GLN	156	A	<-->	9083	O	TYR	326	B	2.83
42.	1376	NH1	ARG	158	A	<-->	9065	O	GLY	323	B	2.69
43.	1376	NH1	ARG	158	A	<-->	9070	O	GLY	324	B	2.73
44.	1377	NH2	ARG	158	A	<-->	9070	O	GLY	324	B	2.75
45.	1506	OG	SER	171	A	<-->	8519	N	GLY	262	B	2.85
46.	1588	O	ASN	180	A	<-->	9187	NH1	ARG	338	B	2.67
47.	1832	O	ASN	206	A	<-->	9188	NH2	ARG	338	B	2.62

Salt bridges

	TLR5 ATOM Atom no.	Atom name	Res name	Res no.	Chain		Vaccine ATOM Atom no.	Atom name	Res name	Res no.	Chain	Distance
1.	59	OD2	ASP	27	A	<-->	8655	ND1	HIS	277	B	2.97

Table S17. List of atom-atom interactions across vaccine-TLR3 interface.

Hydrogen bonds

	TLR3 ATOM Atom no.	Atom name	Res name	Res no.	Chain		Vaccine ATOM Atom no.	Atom name	Res name	Res no.	Chain	Distance
1.	153	NZ	LYS	41	A	<-->	10074	O	GLY	371	B	2.50
2.	4254	OE1	GLU	460	A	<-->	9568	NH1	ARG	316	B	2.72
3.	4277	OH	TYR	462	A	<-->	9568	NH1	ARG	316	B	2.79
4.	4277	OH	TYR	462	A	<-->	9569	NH2	ARG	316	B	2.63
5.	4834	OD1	ASN	517	A	<-->	9766	NH2	ARG	338	B	2.77
6.	5049	O	ASN	540	A	<-->	9765	NH1	ARG	338	B	2.89
7.	5049	O	ASN	540	A	<-->	9766	NH2	ARG	338	B	2.74
8.	5354	O	SER	571	A	<-->	9765	NH1	ARG	338	B	3.21
9.	5603	OD1	ASN	597	A	<-->	9738	N	GLY	335	B	2.97
10.	5602	ND2	ASN	597	A	<-->	9746	O	GLY	336	B	2.79
11.	5663	OG	SER	604	A	<-->	9107	N	GLY	264	B	3.03
12.	5692	ND2	ASN	607	A	<-->	9100	O	GLY	262	B	3.17
13.	5802	NE2	GLN	618	A	<-->	9643	O	GLY	323	B	3.19
14.	5815	NZ	LYS	619	A	<-->	9643	O	GLY	323	B	2.57
15.	5815	NZ	LYS	619	A	<-->	9648	O	GLY	324	B	2.75
16.	5905	NZ	LYS	628	A	<-->	9145	OH	TYR	268	B	2.63
17.	6032	OD2	ASP	641	A	<-->	9655	OG	SER	325	B	3.15
18.	6258	OE2	GLU	663	A	<-->	8985	NH2	ARG	252	B	2.73
19.	6428	N	HIS	682	A	<-->	9286	O	GLU	284	B	3.07
20.	6435	ND1	HIS	682	A	<-->	9316	N	HIS	288	B	3.34
21.	6501	O	ARG	689	A	<-->	9380	NE2	GLN	294	B	2.93
22.	6507	NH1	ARG	689	A	<-->	9376	O	GLN	294	B	2.75
23.	6507	NH1	ARG	689	A	<-->	9388	O	VAL	295	B	2.69
24.	6508	NH2	ARG	689	A	<-->	9381	OE1	GLN	294	B	3.00
25.	6518	O	LEU	690	A	<-->	9359	NZ	LYS	292	B	2.71

Salt bridges

	TLR3 ATOM Atom no.	Atom name	Res name	Res no.	Chain		Vaccine ATOM Atom no.	Atom name	Res name	Res no.	Chain	Distance
1.	4255	OE2	GLU	460	A	<-->	9568	NH1	ARG	316	B	2.72
2.	6257	OE1	GLU	663	A	<-->	8985	NH2	ARG	252	B	2.73

Table S18. List of atom-atom interactions across vaccine-TLR7 interface.

Hydrogen bonds

	<----- TLR7 ATOM ----->						<----- Vaccine ATOM ----->					
	**Atom no.**	**Atom name**	**Res name**	**Res no.**	**Chain**		**Atom no.**	**Atom name**	**Res name**	**Res no.**	**Chain**	**Distance**
1.	10	NH1	ARG	28	A	<-->	8401	OG	SER	47	B	2.68
2.	10	NH1	ARG	28	A	<-->	8544	OG1	THR	62	B	2.88
3.	49	O	PRO	31	A	<-->	10642	N	GLY	282	B	2.47
4.	105	OD2	ASP	37	A	<-->	10555	NZ	LYS	272	B	2.57
5.	107	N	VAL	38	A	<-->	10631	O	MET	280	B	2.97
6.	121	OG1	THR	39	A	<-->	10541	O	LEU	271	B	2.83
7.	127	O	LEU	40	A	<-->	10586	ND1	HIS	275	B	2.95
8.	225	OD2	ASP	50	A	<-->	10555	NZ	LYS	272	B	2.64
9.	546	ND1	HIS	86	A	<-->	10710	O	ASP	290	B	2.82
10.	560	NH1	ARG	87	A	<-->	10668	O	GLY	285	B	2.62
11.	560	NH1	ARG	87	A	<-->	10673	O	LEU	286	B	2.66
12.	665	NH2	ARG	97	A	<-->	10517	OH	TYR	268	B	2.85
13.	781	OD1	ASN	110	A	<-->	11115	N	GLY	336	B	2.86
14.	780	ND2	ASN	110	A	<-->	11104	O	ILE	334	B	3.30
15.	788	O	MET	111	A	<-->	11115	N	GLY	336	B	3.08
16.	818	NZ	LYS	114	A	<-->	11047	O	LEU	327	B	2.54
17.	878	NZ	LYS	119	A	<-->	11056	O	ILE	328	B	2.73
18.	930	O	SER	124	A	<-->	10745	N	GLN	294	B	2.95
19.	969	OH	TYR	128	A	<-->	10310	NZ	LYS	248	B	2.61
20.	1097	OE2	GLU	141	A	<-->	11022	N	SER	325	B	2.91
21.	1305	NH2	ARG	164	A	<-->	10986	O	LEU	320	B	2.79
22.	1305	NH2	ARG	164	A	<-->	10999	OD1	ASN	321	B	2.67
23.	1320	NZ	LYS	165	A	<-->	10808	OG	SER	301	B	2.64
24.	1320	NZ	LYS	165	A	<-->	10814	O	MET	302	B	2.67
25.	1644	NZ	LYS	197	A	<-->	10831	O	LEU	304	B	2.59
26.	1644	NZ	LYS	197	A	<-->	10856	NE2	HIS	306	B	2.85
27.	1652	O	ASP	198	A	<-->	10808	OG	SER	301	B	2.61
28.	1656	OD2	ASP	198	A	<-->	10828	N	LEU	304	B	2.80
29.	1692	OD1	ASN	202	A	<-->	10811	N	MET	302	B	2.87
30.	1691	ND2	ASN	202	A	<-->	10796	O	GLY	299	B	2.83
31.	1930	OG1	THR	228	A	<-->	10413	NZ	LYS	257	B	2.63
32.	1941	OE2	GLU	229	A	<-->	10435	NH1	ARG	259	B	2.80
33.	2822	OE1	GLU	318	A	<-->	10461	NH1	ARG	261	B	2.67
34.	4394	O	ARG	476	A	<-->	10517	OH	TYR	268	B	2.65
35.	4439	ND2	ASN	479	A	<-->	10482	O	GLY	264	B	2.92
36.	4476	O	SER	483	A	<-->	10474	N	GLY	263	B	2.97
37.	4478	OG	SER	483	A	<-->	10455	O	ARG	261	B	2.76
38.	4478	OG	SER	483	A	<-->	10459	NE	ARG	261	B	2.96

Salt bridges

	<----- TLR7 ATOM ----->						<----- Vaccine ATOM ----->					
	**Atom no.**	**Atom name**	**Res name**	**Res no.**	**Chain**		**Atom no.**	**Atom name**	**Res name**	**Res no.**	**Chain**	**Distance**
1.	105	OD2	ASP	37	A	<-->	10555	NZ	LYS	272	B	2.57
2.	225	OD2	ASP	50	A	<-->	10555	NZ	LYS	272	B	2.64

3.	584	OD2	ASP	89	A	<-->	10731	NZ	LYS	292	B	2.60
4.	1940	OE1	GLU	229	A	<-->	10413	NZ	LYS	257	B	2.65
5.	1940	OE1	GLU	229	A	<-->	10436	NH2	ARG	259	B	2.77
6.	2823	OE2	GLU	318	A	<-->	10461	NH1	ARG	261	B	2.67

Table S19. List of atom-atom interactions across vaccine-TLR8 interface.

Hydrogen bonds

	<----- TLR8 ATOM ----->						<----- Vaccine ATOM ----->					
	**Atom no.**	**Atom name**	**Res name**	**Res no.**	**Chain**		**Atom no.**	**Atom name**	**Res name**	**Res no.**	**Chain**	**Distance**
1.	415	OD1	ASP	72	A	<-->	11045	OG1	THR	345	B	3.03
2.	1544	O	CYS	187	A	<-->	10487	OG	SER	283	B	2.77
3.	1986	OE1	GLN	233	A	<-->	10472	N	GLY	281	B	2.84
4.	1985	NE2	GLN	233	A	<-->	10480	O	GLY	282	B	2.78
5.	2007	NZ	LYS	235	A	<-->	10466	O	MET	280	B	2.59
6.	2620	NE	ARG	300	A	<-->	10352	OH	TYR	268	B	2.87
7.	2623	NH2	ARG	300	A	<-->	10352	OH	TYR	268	B	2.73
8.	2638	NZ	LYS	301	A	<-->	10312	O	GLY	263	B	2.62
9.	2638	NZ	LYS	301	A	<-->	10324	OG	SER	265	B	2.62
10.	2820	OE2	GLU	319	A	<-->	10950	N	GLY	336	B	2.81
11.	3372	NH1	ARG	375	A	<-->	10953	O	GLY	336	B	2.87
12.	3892	OG	SER	426	A	<-->	10972	NH1	ARG	338	B	2.77
13.	3925	NH1	ARG	429	A	<-->	10844	SD	MET	322	B	3.21
14.	3926	NH2	ARG	429	A	<-->	10844	SD	MET	322	B	3.22
15.	4153	O	ILE	451	A	<-->	10865	N	TYR	326	B	3.05
16.	4166	NE	ARG	452	A	<-->	10850	O	GLY	323	B	2.75
17.	4169	NH2	ARG	452	A	<-->	10834	OD1	ASN	321	B	3.29
18.	4169	NH2	ARG	452	A	<-->	10850	O	GLY	323	B	2.71
19.	4176	N	LYS	453	A	<-->	10868	O	TYR	326	B	2.60
20.	4196	NE	ARG	454	A	<-->	10926	SG	CYS	332	B	3.03
21.	4198	NH1	ARG	454	A	<-->	10915	O	ASP	331	B	2.70
22.	4199	NH2	ARG	454	A	<-->	10931	O	SER	333	B	2.68
23.	4199	NH2	ARG	454	A	<-->	10933	OG	SER	333	B	2.76
24.	4213	NE	ARG	455	A	<-->	10876	OH	TYR	326	B	3.30
25.	4215	NH1	ARG	455	A	<-->	11158	O	LEU	358	B	2.65
26.	4215	NH1	ARG	455	A	<-->	11167	O	GLY	359	B	2.76
27.	4216	NH2	ARG	455	A	<-->	10876	OH	TYR	326	B	2.72
28.	4268	OE1	GLU	460	A	<-->	11009	SG	CYS	341	B	2.96
29.	4268	OE1	GLU	460	A	<-->	11011	N	LEU	342	B	2.98
30.	4269	OE2	GLU	460	A	<-->	11029	N	GLU	344	B	3.18
31.	4316	OG	SER	465	A	<-->	10994	NE	ARG	340	B	3.15
32.	4316	OG	SER	465	A	<-->	10997	NH2	ARG	340	B	2.60
33.	4322	O	ASN	466	A	<-->	10996	NH1	ARG	340	B	2.55
34.	4322	O	ASN	466	A	<-->	10997	NH2	ARG	340	B	2.87
35.	4333	O	PHE	467	A	<-->	10936	N	ILE	334	B	2.83
36.	4359	O	HIS	469	A	<-->	10996	NH1	ARG	340	B	2.57
37.	4368	N	PHE	470	A	<-->	10948	O	GLY	335	B	2.81
38.	4392	O	ARG	472	A	<-->	10960	OG	SER	337	B	2.71
39.	4398	NH1	ARG	472	A	<-->	10983	O	PRO	339	B	2.71
40.	4399	NH2	ARG	472	A	<-->	11007	O	CYS	341	B	2.71
41.	4416	O	LEU	474	A	<-->	10970	NE	ARG	338	B	2.88
42.	4416	O	LEU	474	A	<-->	10973	NH2	ARG	338	B	2.86
43.	4536	OD2	ASP	487	A	<-->	10973	NH2	ARG	338	B	2.67
44.	4580	OG	SER	492	A	<-->	10827	N	ASN	321	B	2.89
45.	5036	NH1	ARG	541	A	<-->	11177	O	SER	361	B	2.64
46.	5036	NH1	ARG	541	A	<-->	11200	OE1	GLN	363	B	3.01
47.	5037	NH2	ARG	541	A	<-->	11200	OE1	GLN	363	B	2.69
48.	5241	OH	TYR	563	A	<-->	11145	NE	ARG	357	B	2.88

49.	5311	NH2	ARG	569	A	<-->	11190	OE2	GLU	362	B	2.73
50.	5550	NE2	HIS	593	A	<-->	11148	NH2	ARG	357	B	2.86
51.	7592	NH1	ARG	797	A	<-->	11060	O	GLY	347	B	2.68
52.	7593	NH2	ARG	797	A	<-->	11060	O	GLY	347	B	2.91

Salt bridges

	<----- **TLR8 ATOM** ----->						<----- **Vaccine ATOM** ----->					
	**Atom no.**	**Atom name**	**Res name**	**Res no.**	**Chain**		**Atom no.**	**Atom name**	**Res name**	**Res no.**	**Chain**	**Distance**
1.	4536	OD2	ASP	487	A	<-->	10973	NH2	ARG	338	B	2.67
2.	5310	NH1	ARG	569	A	<-->	11190	OE2	GLU	362	B	2.68
